# *MdARF3* switches the lateral root elongation to regulate dwarfing in apple plants

**DOI:** 10.1093/hr/uhae051

**Published:** 2024-02-23

**Authors:** Jiahong Lv, Yi Feng, Longmei Zhai, Lizhong Jiang, Yue Wu, Yimei Huang, Runqi Yu, Ting Wu, Xinzhong Zhang, Yi Wang, Zhenhai Han

**Affiliations:** Institute for Horticultural Plants, China Agricultural University, Beijing 100193, China; Key Laboratory of Biology and Genetic Improvement of Horticultural Crops (Nutrition and Physiology), Ministry of Agriculture and Rural Affairs, Beijing 100193, China; Institute for Horticultural Plants, China Agricultural University, Beijing 100193, China; Key Laboratory of Biology and Genetic Improvement of Horticultural Crops (Nutrition and Physiology), Ministry of Agriculture and Rural Affairs, Beijing 100193, China; Institute for Horticultural Plants, China Agricultural University, Beijing 100193, China; Key Laboratory of Biology and Genetic Improvement of Horticultural Crops (Nutrition and Physiology), Ministry of Agriculture and Rural Affairs, Beijing 100193, China; Institute for Horticultural Plants, China Agricultural University, Beijing 100193, China; Key Laboratory of Biology and Genetic Improvement of Horticultural Crops (Nutrition and Physiology), Ministry of Agriculture and Rural Affairs, Beijing 100193, China; Institute for Horticultural Plants, China Agricultural University, Beijing 100193, China; Key Laboratory of Biology and Genetic Improvement of Horticultural Crops (Nutrition and Physiology), Ministry of Agriculture and Rural Affairs, Beijing 100193, China; Institute for Horticultural Plants, China Agricultural University, Beijing 100193, China; Key Laboratory of Biology and Genetic Improvement of Horticultural Crops (Nutrition and Physiology), Ministry of Agriculture and Rural Affairs, Beijing 100193, China; Institute for Horticultural Plants, China Agricultural University, Beijing 100193, China; Key Laboratory of Biology and Genetic Improvement of Horticultural Crops (Nutrition and Physiology), Ministry of Agriculture and Rural Affairs, Beijing 100193, China; Institute for Horticultural Plants, China Agricultural University, Beijing 100193, China; Key Laboratory of Biology and Genetic Improvement of Horticultural Crops (Nutrition and Physiology), Ministry of Agriculture and Rural Affairs, Beijing 100193, China; Institute for Horticultural Plants, China Agricultural University, Beijing 100193, China; Key Laboratory of Biology and Genetic Improvement of Horticultural Crops (Nutrition and Physiology), Ministry of Agriculture and Rural Affairs, Beijing 100193, China; Institute for Horticultural Plants, China Agricultural University, Beijing 100193, China; Key Laboratory of Biology and Genetic Improvement of Horticultural Crops (Nutrition and Physiology), Ministry of Agriculture and Rural Affairs, Beijing 100193, China; Institute for Horticultural Plants, China Agricultural University, Beijing 100193, China; Key Laboratory of Biology and Genetic Improvement of Horticultural Crops (Nutrition and Physiology), Ministry of Agriculture and Rural Affairs, Beijing 100193, China

## Abstract

Apple rootstock dwarfing and dense planting are common practices in apple farming. However, the dwarfing mechanisms are not understood. In our study, the expression of *MdARF3* in the root system of dwarfing rootstock ‘M9’ was lower than in the vigorous rootstock from *Malus micromalus* due to the deletion of the WUSATAg element in the promoter of the ‘M9’ genotype. Notably, this deletion variation was significantly associated with dwarfing rootstocks. Subsequently, transgenic tobacco (*Nicotiana tabacum*) cv. Xanthi was generated with the ARF3 promoter from ‘M9’ and M. *micromalus* genotypes. The transgenic apple with 35S::MdARF3 was also obtained. The transgenic tobacco and apple with the highly expressed ARF3 had a longer root system and a higher plant height phenotype. Furthermore, the yeast one-hybrid, luciferase, electrophoretic mobility shift assays, and Chip-qPCR identified *MdWOX4-1* in apples that interacted with the pMm-ARF3 promoter but not the pM9-ARF3 promoter. Notably, *MdWOX4-1* significantly increased the transcriptional activity of *MdARF3* and *MdLBD16-2*. However, *MdARF3* significantly decreased the transcriptional activity of *MdLBD16-2*. Further analysis revealed that *MdARF3* and *MdLBD16-2* were temporally expressed during different stages of lateral root development. *pMdLBD16-2* was mainly expressed during the early stage of lateral root development, which promoted lateral root production. On the contrary, *pMmARF3* was expressed during the late stage of lateral root development to promote elongation. The findings in our study will shed light on the genetic causes of apple plant dwarfism and provide strategies for molecular breeding of dwarfing apple rootstocks.

## Introduction

Dwarfing rootstock is extensively utilized in apple cultivation due to its numerous advantages, including early fruit production, high yields, excellent fruit quality and compatibility with machinery, saving on the labor costs. However, the development of novel dwarfing apple rootstocks poses significant challenges and requires a considerable amount of time. For example, previous breeding techniques required the progeny to undergo extensive selection for pest and disease resistance and good performance in the nursery and orchard. As a result, it took 25 to 30 years to commercially release a new hybrid rootstock [1, 2].

Dwarfing rootstocks effectively limit tree size and exert a significant influence on the growth and development of grafted apple varieties [3]. The breeding of superior dwarfing rootstocks stands as a primary objective for apple breeding experts in order to meet the demands of modern apple production. Molecular breeding has emerged as one of the key approaches in contemporary breeding practices to obtain dwarfing rootstocks. However, the molecular mechanism underlying dwarfing induced by rootstocks such as M9 remains unclear, posing a challenge to ongoing breeding efforts [4]. Dwarfing represents a complex trait orchestrated by multiple genes [5]. Recent genetic advancements significantly aid the breeding and selection of novel dwarf apple rootstocks, which has historically depended heavily on experience. Initially, the cross-sectional area of the trunk in an ‘M9’ × Robusta 5 segregation population was used to characterize the phenotypic traits related to dwarfing. Subsequently, bulked segregation analysis preliminarily located the dwarfing segment gene [6]. The genetic distance of this trait is within 2.5 cm at the top of the fifth linkage group of ‘M9’, although there is a great genetic distance between the genetic marker and the target gene. This was the first report on the preliminary location of the dwarfing gene interval on apple rootstocks [6].

With continued research using the dwarfing phenotype data of the isolated population, two dwarfing quantitative trait locus (QTL) loci, Dw1 and Dw2, were located through genetic mapping and linkage analysis [5]. The physical position of Dw1 is between 4.74 and 7.62 Mb at the top of the fifth linkage group ‘M9’, including three simple sequence repeats marker loci and 547 genes, with a 56.8% contribution rate to dwarfing [5]. Dw1 is the most valuable QTL segment obtained from positive genetic methods [5, 7].

The synthesis, transportation, and degradation of phytohormones are closely related to the dwarfing phenotype of rootstocks. A previous study on the root replacement treatment of dwarfing rootstocks revealed that after the dwarfing rootstock root system was recovered by vigorous rootstocks, the indole acetic acid content in the scions was compensated, and the new shoots resumed growth, implying that the first signal source of dwarfing effect is in the root system of dwarfing rootstocks [8]. Further research revealed that applying 1-napthalene acetic acid to the dwarfing rootstocks did not increase the annual growth of the new shoots, and the growth potential was not recovered, suggesting that the auxin response pathway in dwarfing rootstocks may be blocked [9].

Many terrestrial plant developmental and growth processes, from embryogenesis to aging, depend on the auxin response system. Besides, most plants express genes controlled by auxin, which initiates or mediates the growth and development [10, 11]. The auxin signaling pathway mediated by ARF also controls cell division, lengthening, and specialization, impacting plant progression [12]. Due to the limited number of its auxin signal-specific components, different attributes between ARF families may help establish various unique auxin responses during plant development and growth [11]. In angiosperms, root formation is the foundation for plant growth. In Arabidopsis, AtARF2, 7, and 19 control root formation and growth [13, 14]. *AtARF10* and *16* regulate the formation and growth of root caps in *Arabidopsis thaliana* [15]. Besides, *AtARF11* regulates the hormone signal transmission and growth of lateral roots [16]. In tomatoes (*Solanum lycopersicum* Mill), *SlARF2* also controls the development of lateral roots [17]. Besides, *PpARF14*, *69*, *12*, *14*, and *18* regulate root formation and growth in peaches (*Prunus persica* L. Batsch) [18]. On the other hand, *MiARF2* regulates the initiation and growth of adventitious roots and root primordia [19].

WUSCHEL (WUS)-related Homeobox (WOX) genes are crucial for coordinating gene transcription associated with shoot and root meristem function and organogenesis [20–22]. For example, WOX4 promotes the formation of procambial or cambial stem cells [23]. The cambium is responsible for thickening extended roots and stems [24]. Notably, *A. thaliana* and tomato express WOX4 in the growing vascular bundles of lateral root and shoot organs. In *A. thaliana*, a low expression of WOX4 leads to the developing of tiny plants with severely reduced levels of distinct xylem and phloem [25]. Additionally, a WOX family member is necessary for the auxin-dependent regulation of lateral plant growth due to the similarities between the control of plant apical meristem and lateral growth [26]. The auxin-induced development of lateral roots is mostly mediated by *AtLBD16*, activated by the auxin response factors ARF7 and ARF19, which control the production of lateral roots [26, 27]. Furthermore, LBD18 and LBD16, located downstream of ARF7 and ARF19, also control the development of lateral roots [28]. LBD16 also promotes cell division during the start of a callus. According to Liu *et al.* [29], WOX11 regulates LBD16 to promote the acquisition of cellular multipotency in calli tissues. However, the relationship between ARF, WOX, and LBD in lateral root generation and plant growth remains unclear.

This study observed a deletion variation in the dwarfing rootstock M9 promoter compared to *Malus micromalus*, implying that the apple plant growth is differentially regulated. Besides, a WOX-ARF-LBD module regulated the plant dwarfing traits by promoting lateral root development. The findings in our study offer a valuable theoretical basis for understanding the molecular mechanisms that underlie the regulation of apple growth.

## Results

### ARF3 on the Dw1 loci was differentially expressed between vigorous and dwarfing rootstocks

**Figure 1:**
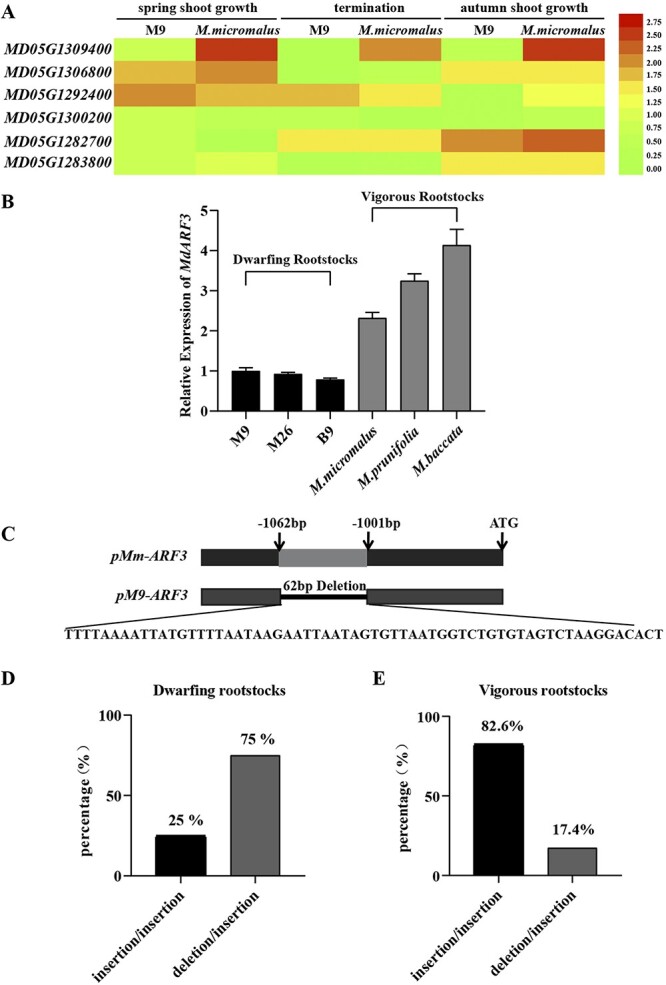
*MdARF3* expression on the Dw1 loci between vigorous and dwarfing rootstocks. **A** Differentially expressed hormone-related genes in DW1 between M9 and *Malus micromalus* at different developmental stages. **B***MdARF3* expression in the roots of different vigorous and dwarfing rootstocks. **C** Schematic diagram of the ARF3 promoter in M9 and *M. micromalus*. **D** The variation rate of dwarfing rootstocks lacking M9 in either parent. **E** The variation rate of vigorous rootstocks. ARF, auxin response factor. Red represents higher expression, and green represents weaker expression.

Six genes in the QTL segments that were related to plant hormones were screened based on the published QTL segments of apple rootstock dwarfing traits [5] (Table S2, see online supplementary material). We examined the expression of these genes in the root of rootstocks at three different growth periods of the apple plant, including the stage of spring shoot growth, termination, and autumn shoot growth. The results showed that only *Md05G1309400* was consistently expressed at significantly lower levels in M9 than that in *M. micromalus*, while the expression of other genes, including *MD05G1306800*, *MD05G1292400*, *MD05G1300200*, *MD05G1282700*, and *MD05G1283800* was unstable (Fig. 1A). The evolutionary tree with *Md05G1309400* and the ARF family genes of *A. thaliana* revealed that *Md05G1309400* had the highest homology with *AtARF3*. Thus, it was named *MdARF3* (Auxin response factor 3) (Fig. S1, see online supplementary material). Further analysis revealed a strong differential expression trend of *MdARF3* between vigorous and dwarfing rootstocks. The expression of MdARF3 in the roots of dwarfing rootstocks M9, M26, and B9 was lower than that of vigorous rootstocks *Malusmicromalus*, *Malusprunifolia*, and *Malusbaccata* (Fig. 1B). This result showed that the dwarfing phenotypic may be connected to the low *MdARF3* expression. To comprehend the causes of variations in *MdARF3* expression levels, the ARF3 upstream promoter sequence was cloned. The ARF3 promoter sequence in M9 (pM9-ARF3) had a 62 bp deletion sequence (−TTTTAAAATTATGTTTTAATAAGAATTAATAGTGTTAATGGTCTGTGTAGTCTAAGGACACT-) and 10 single nucleotide polymorphism (SNP) sites compared to *M. micromalus* (Fig. 1C) implying that the variations might be related to the *MdARF3* expression.

Correlation analysis of the 62 bp deletion sequence between dwarfing and vigorous rootstocks using an InDel gene marker for the *MdARF3* gene had a deletion heterozygous variation in ‘M9’, with a deletion/insertion genotype. However, *M. micromalus* had a homozygous insertion with an insertion/insertion genotype. All comparisons between the dwarfing and vigorous rootstocks revealed that the dwarfing rootstocks had the deletion/insertion genotype, where the parent contained M9 (Table S3, see online supplementary material). Besides, where the parent did not contain M9, the deletion/insertion genotype also reached 75% in 24 dwarfing rootstocks (Fig. 1D; Table S3, see online supplementary material). In 23 vigorous rootstocks, the insertion/insertion genotype reached 82.6% (Fig. 1E; Table S3, see online supplementary material), implying that the 62 bp deletion variation in the ARF3 promoter was highly linked to the rootstock type.

### A WUS element deletion on the ARF3 promoter reduced its transcriptional activity

**Figure 2:**
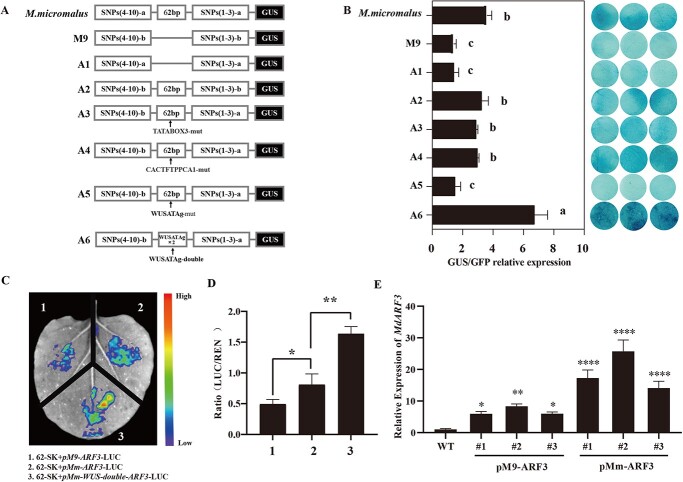
The transcriptional activity of the ARF3 promoter is diminished due to the lack of the WUS element. **A** Symbolic representation of the GUS ARF3 promoter. **B** Relative expression of GUS/GFP in GUS-stained leaves of *Nicotiana tabacum cv. Xanthi* following a brief co-expression of various ARF3: GUS promoters with pro35S::GFP. **C** Imaging analysis of the dual-luciferase reporter system. Violet tint denotes a weaker signal, and red a stronger signal. **D** ARF3 promoter fluorescence in tobacco leaves and the LUC/REN ratio. **E** Relative expression of *MdARF3* in the WT, pM9-ARF3 transgenic tobacco, and pMm-ARF3 transgenic tobacco T1 generation seedlings. ^*^P < 0.05; ^**^P < 0.01; ^****^P < 0.0001. GFP, green fluorescent protein; GUS, β-glucuronidase; WT, wild type.

To determine if the promoter sequence variations impact ARF3 expression, GUS staining was performed. The relative level of GUS/GFP expression induced by pM9-ARF3 was much lower than that of pMm-ARF3 (Fig. 2A and B). Besides, after deleting the 62 bp on the pMm-ARF3 promoter (A1), the promoter staining and GUS/GFP activity were significantly reduced, though not significantly different from the pM9-ARF3 promoter activity (Fig. 2A and B). On the contrary, the insertion of the 62 bp sequence into the pM9-ARF3 promoter (A2) significantly increased the promoter activity, with no discernible distinction compared to the pMm-ARF3 activity (Fig. 2A and B). These findings indicate that the 62 bp deletion sequence in the pM9-ARF3 promoter weakened the pM9-ARF3 activity.

PLACE predictions identified three elements in the 62 bp deletion promoter, including TATABOX3, CACTFTPPCA1, and WUSATAg (Table S4, see online supplementary material). The three elements on the pMm-ARF3 promoter were mutated and labeled A3, A4, and A5 (Fig. 2A), respectively. Notably, the WUSATAg element mutation (A5) considerably reduced the ARF3 promoter activity, even though there was no apparent distinction in the activity between A3 and A4 promoters compared to the *M. micromalus* promoter. Compared to the A2 theory, only one copy of the TTAATGG (WUSATAg) sequence did not change the GUS expression. However, TTAATGG sequences placed consecutively with the A6 construct considerably increased promoter activation. (Fig. 2A and B).

Validation of the luciferase (LUC) activity in different promoters using a dual-LUC reporting system revealed that the pMm-ARF3 promoter LUC activity was substantially greater than the pM9-ARF3 promoter LUC activity. Furthermore, doubling the WUSATAg element obtained the pMm-WUS-double-ARF3 promoter, which LUC activity was significantly higher than that of pMm-ARF3 (Fig. 2C and D). Analysis of the stable conversion of ARF3 into tobacco with different promoters, including pM9-ARF3-101eGFP (pM9-ARF3) and pMm-ARF3-101eGFP (pMm-ARF3) revealed that the relative degree of *MdARF3* expression in the pMm-ARF3 transgenic tobacco was higher than in the pM9-ARF3 transgenic tobacco (Fig. 2E).

Together, these findings indicate that the deletion in the MdARF3 promoter weakens its basal activity, and the presence or absence of the WUSATAg element is the primary reason for the difference in the MdARF3 promoter activity.

### ARF3 promotes root elongation and plant growth in transgenic tobacco

**Figure 3:**
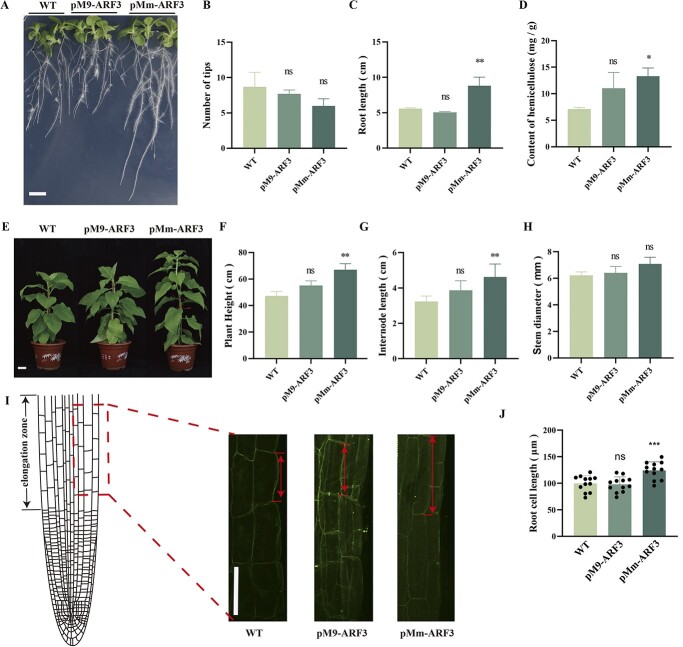
ARF3 enhances aboveground plant growth by stimulating root elongation. **A** Plant phenotypes; **B** the number of root tips; **C** the root length; and **D** the quantity of hemicellulose in WT, pM9-ARF3, and pMm-ARF3 transgenic tobacco (*Nicotiana tabacum cv. Xanthi*) T1 generation seedlings after 15 days in the MS medium. Sizing guide: 1 cm. **E** The plant phenotypes; **F** the tobacco plant height; **G** internode length; and **H** stem diameter of WT, pM9-ARF3, and pMm-ARF3 transgenic tobacco 30 days after transplantation into the soil. Scale bar: 5 cm. **I** Root longitudinal sections; and **J** cell length at the elongation region in ARF3 transgenic plants. Scale bar: 100 μm. Data represent the average ± SD of three separate tests. ns denotes no significant difference; SD indicates standard deviation; WT denotes wild type. ^*^P < 0.05; ^**^P < 0.01.

To confirm the function of ARF3, its ectopic expression in tobacco plants was introduced under the *M. micromalus* (pMm-ARF3) and M9 (pM9-ARF3) genotype promoters (Figs 2E and 3). The pMm-ARF3 genotype root length was considerably greater than in the pM9-ARF3 and WT tobacco genotypes after 15 days in the medium (Fig. 3A and C). Comparisons of the composition of the three main cell wall substances, including pectin, cellulose, and hemicellulose, among the WT, pM9-ARF3, and pMm-ARF3 transgenic tobacco roots revealed that only the hemicellulose content of the pMm-ARF3 was considerably higher than in WT and pM9-ARF3 (Fig. 3D; Fig. S2, see online supplementary material). Moreover, after being transplanted into the soil for 30 days, the genetically modified plant pMm-ARF3 showed significantly increased plant height and internode length compared to the wild type. There was no difference observed between pM9-ARF3 and the wild type. (Fig. 3E–G). However, there was no discernible change in the number of root nodes, tips, or stem diameter among the three genotypes (Figs 3B and H; Fig. S3, see online supplementary material). Additionally, the cell length of the elongation zone in pMm-ARF3 transgenic plants was significantly high compared to WT and pM9-ARF3 (Fig. 3I and J). In summary, the high *MdARF3* expression level promoted root elongation and the aboveground growth of tobacco plants.

### ARF3 promotes root elongation and plant growth in transgenic apple

**Figure 4:**
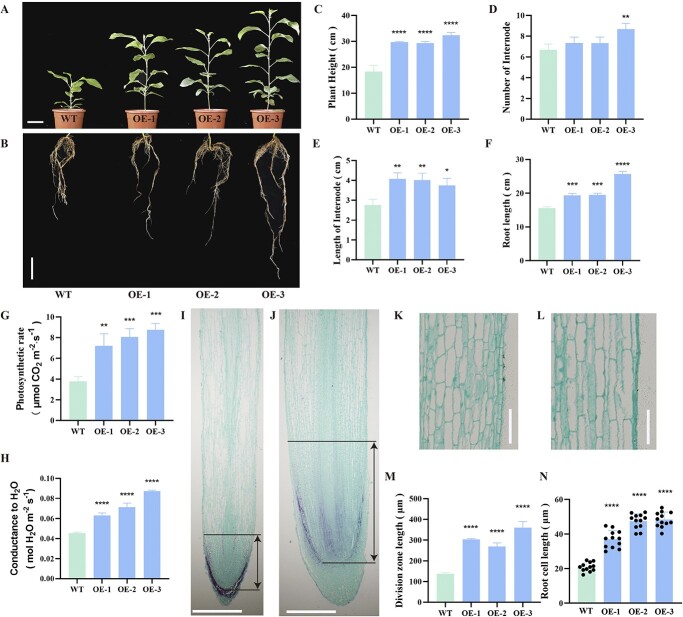
MdARF3 promotes apple plant growth through root elongation. **A** The plant and **B** root phenotypes and length of WT, and 35S::MdARF3 (OE) transgenic apple plants 120 days after transplantation into the soil. Scale bar: 5 cm. **C** The plant height; **D** number of internode; **E** length of internode; **F** root length; **G** photosynthetic rate; and **H** intercellular CO_2_ concentration in WT, and 35S::MdARF3 transgenic apple plants. The root tip region of **I** WT and **J** 35S::MdARF3 transgenic apple plants. Scale bar: 100 μm. The root elongation sections in **K** WT and **L** 35S::MdARF3 transgenic apple plants. Scale bar: 100 μm. **M** The root division zone and **N** cell lengths. ^*^P < 0.05; ^**^P < 0.01; ^***^P < 0.001; ^****^P < 0.0001.

Golden Delicious apple leaf explants transformation yielded 35:: MdARF3 transgenic positive line (Fig. S4, see online supplementary material). Phenotypical characterization between the WT and 35:: MdARF3 transgenic apples plants on the soil for 120 days revealed that 35::MdARF3 had a significant height and root length growth compared to WT (Fig. 4A–C and F). Although the number of internodes was not significantly different between the WT and 35::MdARF3 transgenic apple plants, the internode length was increased in the 35::MdARF3 transgenic apple plants (Fig. 4D and E). Physiological parameters such as photosynthetic rate and intercellular CO_2_ concentration were also measured. The photosynthetic rate and intercellular CO_2_ concentration in WT were also significantly lower than 35S::MdARF3 transgenic apple (Fig. 4G and H). Furthermore, the root longitudinal segment analysis revealed that the length of WT division zone was significantly shorter than in 35S::MdARF3 transgenic apple (Fig. 4I, J, M). Besides, the length of elongation root region cells in 35::MdARF3 were longer than those in WT (Fig. 4K, L, N). Taken together, the apple plant root elongation and the development of aboveground organs are promoted by *MdARF3* expression.

### MdWOX4–1 positively controls the ARF3 expression by directly binding the WUSATAg element

**Figure 5:**
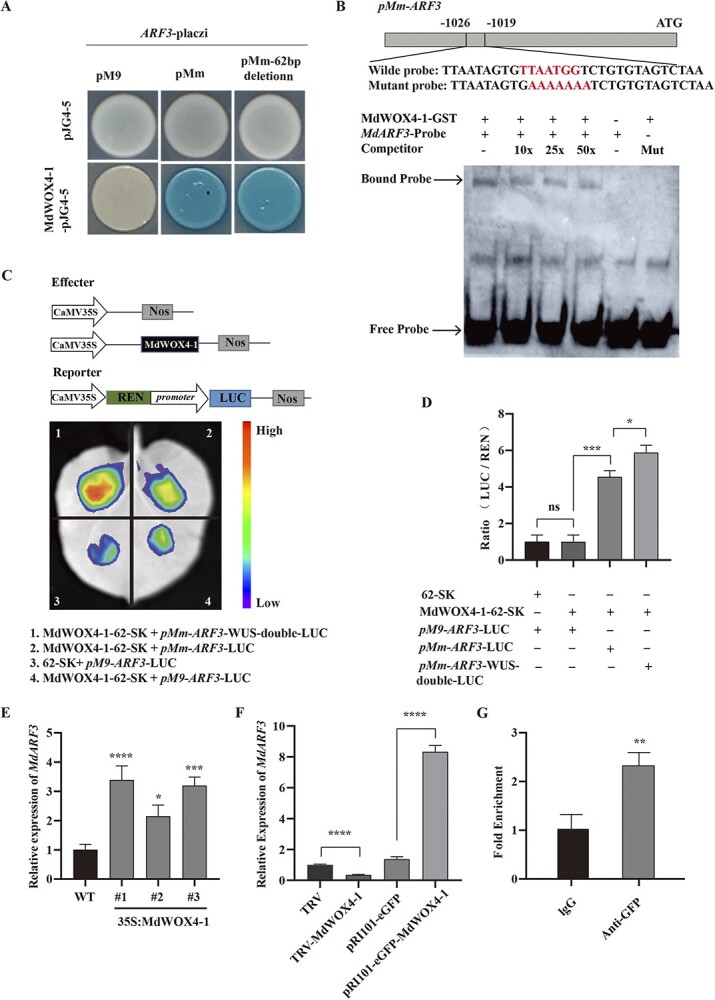
MdWOX4–1 binds the WUSATAg element *in vivo* and *in vitro*. **A** MdWOX4–1 transcriptional activation in yeast cells. **B** MdWOX4–1 binding the ARF3 promoter based on the EMSA assay. ‘-’ indicates not present and ‘+’ indicates that the corresponding proteins or probes are present. ‘10x’, ‘25x’, and ‘50x’ indicates unlabeled probes employed as competitors. **C** Schematic representation of the constructs used to prepare effector and reporter strains in the dual-luciferase (LUC) assay and analysis of the dual-luciferase imaging. Stronger and weak signals are represented by red and violet, respectively. **D** The LUC/REN ratio and the effect of MdWOX4–1 on the ARF3 promoter in tobacco leaves. **E** Relative expression of *MdARF3* expression in *MdWOX4* transgenic calli. **F** Relative level of *MdARF3* expression in pRI101-eGFP-MdWOX4–1 and TRV-MdWOX4–1 transgenic apple roots. **G** ChIP-qPCR analysis. Error bars indicate standard deviations. ns, no significant difference; EMSA, electrophoretic mobility shift assay. ^*^P < 0.05; ^**^P < 0.01; ^***^P < 0.001; ^****^P < 0.0001.

Studies have shown that the WUSATAg element (TTAATGG) is the binding element of WOX genes [30, 31]. A total of 18 MdWOX genes were previously identified in apples [32]. Y1-H test revealed that the genes MdWOX4–1, MdWOX11/12–2, and MdWOX13–1 bind the pMm-ARF3 promoter, containing the 62 bp sequence, but not the pM9-ARF3 promoter lacking the 62 bp sequence (Fig. 5A; Fig. S5, see online supplementary material). Moreover, the GUS test showed that MdWOX4-1 and MdWOX13-1 promote the pMmARF3 promoter activity (Fig. S6, see online supplementary material). In addition, EMSA revealed that the MdWOX4-1 and MdWOX13-1 proteins strongly bind to the biotin-labeled ARF3 probe (Fig. 5B; Fig. S7A, see online supplementary material). The LUC test also revealed that MdWOX4-1 and MdWOX13-1 promoted the pMmARF3 promoter activity. The reporter constructs were created by cloning pMm-ARF3-WUS-double into the pGreenII 0800-LUC plasmid. Moreover, the LUC/REN ratio significantly improved in the co-transformation of MdWOX4-1 or MdWOX13-1 and the pMm-ARF3-0800 or pMm-ARF3-WUS-double-0800 promoters compared to the pM9-ARF3-0800 co-transformation (Fig. 5C and D; Fig. S7B–D, see online supplementary material).

The MdWOX4-1 and MdWOX13-1 expressions were also induced in apple callus ‘Orin’ (*Malus domestica* Borkh) using the constitutive CaMV35S promoter (Figs S8 and S9A, see online supplementary material). The relative *MdARF3* expression level was significantly increased in the three *MdWOX4-1* transgenic lines but not in *MdWOX13-1* (Fig. 5E; Fig. S9B, see online supplementary material). Besides, during the transient expression or inhibition of *MdWOX4-1* or *MdWOX13-1* in the apple seedling root system (*M. baccata*) (Figs S9C and S10, see online supplementary material), the *ARF3* expression was correspondingly increased in the overexpressed lines and decreased in the *MdWOX4-1* silenced lines, but not in *MdWOX13-1* (Fig. 5F; Fig. S9D, see online supplementary material). The ChIp assay also demonstrated that *MdWOX4-1* positively regulates *ARF3 in vivo* (Fig. 5G). These results further revealed that *MdWOX4-1* effectively promoted *ARF3* expression in apple plants.

### MdARF3 directly reduced MdLBD16-2 expression, while MdWOX4-1 directly increased MdLBD16-2 expression

**Figure 6:**
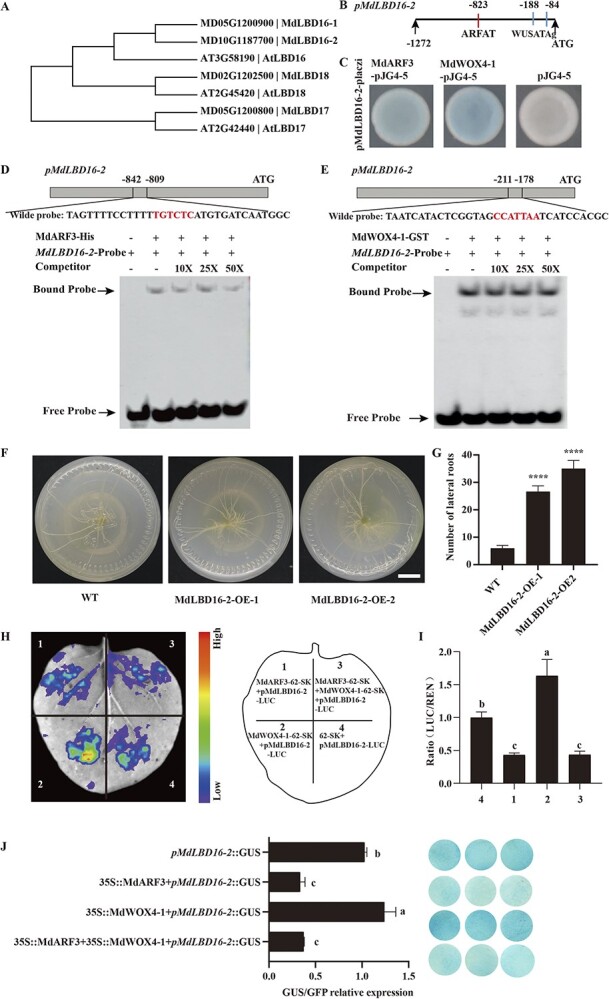
*MdARF3* inhibits *MdLBD16–2* expression and *MdWOX4–1* improves *MdLBD16–2* expression *in vivo* and *in vitro*. **A** The evolutionary relationships among *AtLBD16, AtLBD17, AtLBD18, MdLBD16–1, MdLBD16–2, MdLBD17*, and *MdLBD18*. **B** The schematic diagram of *MdLBD16–2* promoter cis-element. **C** Yeast one-hybrid assay validates the binding interaction between MdWOX4–1 and MdARF3 on the *MdLBD* promoter. **D***MdARF3* and **E***MdWOX4–1* binding the *MdLBD16–2* promoter in EMSA method test. **F** The root phenotypes and **G** number of lateral roots in WT and MdLBD16–2-OE transgenic tobacco plants after 15 days in the medium. Scale bar: 2 cm. **H**–**I** The LUC/REN ratio and the effect of MdARF3 or MdWOX4–1 on the *MdLBD16–2* promoter in tobacco leaves. Red represents a stronger signal, and violet a weaker signal. **J** The relative expression of GUS/GFP and GUS staining in the *Nicotiana tabacum* cv*. Xanthi* leaves following the brief co-expression of pLBD16–2::GUS promoters with pro35S::GFP or 35::MdARF3 and 35S::MdWOX4–1. ^****^P < 0.0001.

*LBD16*, *LBD17*, and *LBD18* positively influence the emergence of lateral roots in *A. thaliana* [28, 33, 34]. A homologous comparison identified four genes, including *MD05G1200900, MD10G1187700, MD02G1202500,* and *MD05G1200800* homologous to LBD in *M. domestica* (Fig. 6A). These four gene expression levels were detected using transformed apple callus ‘Orin’ that expressed *MdARF3* under the constitutive CaMV35S promoter and RNA interference (Fig. S11, see online supplementary material). The results revealed that the LBD expression was considerably inhibited in the overexpression lines and enhanced in the RNA interference lines of *MdARF3* (Figs S12 and S13, see online supplementary material). Furthermore, the promoter element prediction revealed that *MdLBD16–2* and *MdLBD17* contain the TGTCTC AuxREs element that binds the ARF components and the WUSATAg element containing the WOX binding site (Fig. 6B; Fig. S14A, see online supplementary material). At the same time, the Y1H system revealed that neither MdARF3 nor MdWOX4-1 interacted with the MdLBD17 promoter (Fig. S14B, see online supplementary material). Y1H and EMSA analyses also suggested that *MdARF3* and *MdWOX4-1* bound the MdLBD16-2 promoter (Fig. 6C-E).

MdLBD16-2 overexpressing transgenic tobacco was also obtained. The functional study revealed that the number of lateral roots in MdLBD16-2 transgenic tobacco was significantly higher than in the WT (Fig. 6F and G; Fig. S15, see online supplementary material). The LUC/REN test further revealed that single transfection of MdWOX4-1 significantly promoted MdLBD16-2 (Fig. S16B and C, see online supplementary material), and MdARF3 significantly reduced MdLBD16-2 (Fig. S16C and D, see online supplementary material). On the contrary, a single transfection of MdARF3 and co-transfection of MdARF3 + MdWOX4-1 significantly inhibited the LBD16-2 expression (Fig. 6H and I, Fig. S16, see online supplementary material), consistent with the GUS staining results (Fig. 6J), implying that ARF3 plays a decisive role in the MdWOX4-1-MdARF3-MdLBD16-2 regulatory relationship.

### ARF3 may be a molecular switch between lateral root initiation and elongation

**Figure 7:**
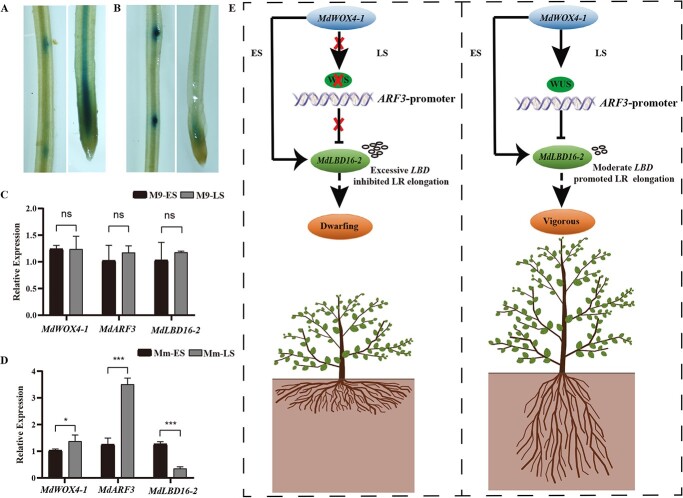
Differential expression of ARF3 and *LBD16–2* during lateral root development and hypothesis model of ARF3 in controlling the dwarfing of apple rootstock. **A** The position of pMmARF3-GUS in the early lateral root stage (ES-before the lateral root emergence) and in the late lateral root stage (LS, the stage of lateral root elongation growth). **B** The position of pMdLBD16–2-GUS during ES and LS. **C** The relative *MdARF3, MdLBD16–2,* and *MdWOX4–1* expressions in the ES and LS of M9. **D** The relative *MdARF3, MdLBD16–2,* and *MdWOX4–1* expressions in the ES and LS of *Malus micromalus*. **E** The proposed model for the regulation of root elongation and plant height in apples by MdWOX4–1-ARF3-LBD16–2. *MdWOX4–1* promotes the expression of *MdLBD16–2* at the initial stage of lateral root development of *M. micromalus*, promoting lateral root development. At the late stage of lateral root development, *MdWOX4–1* promotes the expression of ARF3, thereby suppressing the expression of *MdLBD16–2*, terminating the development of the lateral root, and causing the lateral root to enter the elongation stage. This promotes the arborization of plants. However, in dwarf rootstock, M9, *MdWOX4–1* could not promote the expression of ARF3 or inhibit the expression of *MdLBD16–2* in the LS development of root primordial owing to the lack of the WUS elements on the ARF3 promoter. This resulted in the delayed completion of the lateral root growth process, which inhibited the elongation of the lateral root and resulted in a shorter lateral root, promoting plant dwarfing. Therefore, ARF3 may be a molecular switch between initiating and extending lateral roots. In addition, short lateral roots may be an important reason for the dwarf phenotype of M9. Ns indicates no significant difference. ^*^P < 0.05; ^***^P < 0.001.

Ectopic expression and interaction analysis suggested that *MdWOX4-1* directly positively regulated the transcription of *MdARF3* and *MdLBD16-2*. Interestingly, *MdARF3* directly negatively regulated the transcription of *MdLBD16-2*. Thus, we hypothesized that there might be a temporal specificity of gene regulation in the development of apple roots. To verify this concept, *MdARF3*, and *MdLBD16-2* promoters were constructed onto GUS vectors and *Agrobacterium rhizogenes* used to infect the apple seedling roots (*M. baccata* with the same WUS allele as *M. micromalus* genotype). The results revealed that *MdARF3* was lowly expressed during the early stage of lateral root development (ES, before the LR emergence) and increased in the late stage of lateral root development (LS, the LR elongation growth stage) (Fig. 7A). In contrast, *MdLBD16-2* was highly expressed at ES, and decreased during LS (Fig. 7B). The expression of *MdARF3* and *MdLBD16-2* was further detected in the same position in the *M. micromalus* and M9 seedling roots. The outcomes demonstrated that the degree of *MdARF3* and *MdWOX4-1* expressions was significantly lower during ES than LS, while *MdLBD16-2* was significantly higher in ES than in LS in *M. micromalus* seedlings (Fig. 7D). However, the MdARF3, MdWOX4-1, and MdLBD16-2 expression levels did not differ significantly between the early and late stages in M9 seedlings (Fig. 7C).

These results implied that *MdWOX4-1* may have dual functions. In the prophase stage of root primordia, *MdWOX4-1* promotes the expression of *MdLBD16-2*, thus, promoting the development of lateral roots. At the late stage of root primordia development, *MdWOX4-1* promotes the *MdARF3* expression, thereby blocking the *MdLBD16-2* expression, inhibiting the lateral root development, which pushes the lateral root to the elongation stage.

## Discussion

Dwarfing rootstocks are widely used in modern apple production to achieve high-density planting and efficient mechanical management. The apple dwarfing rootstock ‘Malling9’ (referred to as ‘M9’) has been globally utilized to reduce the growth vigor of grafted trees and serve as a genetic resource for developing new rootstocks, making it crucial to explore its dwarfing mechanism [2, 5].

The utilization of natural variation has enhanced our understanding of phenotypic diversity and trait evolution, ultimately accelerating plant breeding, in which identifying causal variations is crucial [35]. So far, when searching for candidate genes and causal variations, priority has been given to coding sequence variations and transcriptional level polymorphisms caused by promoter variations [36–38]. Foster *et al.* [5] identified a major quantitative trait locus (QTL), Dw1, on linkage group 5 (LG5), which exerts a significant influence on the dwarfing phenotype of the apple scion. In our study, we found that the expression of the MdARF3 which on DW1 in the root system of dwarfing rootstocks lower than that in vigorous rootstocks (Fig. 1B). Furthermore, compared to the M*. micromalus*, there was a 62 bp deletion variation in the promoter region of *MdARF3* in the dwarfing rootstock M9, with a genotype of deletion/insertion. It was verified that this variation was significantly associated with tree dwarfing (Fig. 1D and E; Table S3, see online supplementary material).

Variations in gene sequences due to insertions, deletions, and mutation result in gene functional divergence. In our study, the internode length of pM9-ARF3 transgenic seedlings was significantly shorter than that of the pMm-ARF3 transgenic seedlings, the primary manifestation of the dwarfing phenotype in M9 seedlings. Although a stable transformation phenotype of MdARF3-RNAi was not observed—of which further exploration is needed in the later stage—we conducted a stable transformation experiment using Golden Delicious apple with 35S::MdARF3 and discovered that the overexpression of MdARF3 significantly enhanced internode elongation (Fig. 4E). These findings are consistent with the results reported by Zheng *et al.* [39]. A previous study also reported that M9 reduced the mean length and node number of the ‘Rome Beauty’ primary shoot that regrew after one-year-old scions were trimmed [40]. However, the number of nodes in the present study did not differ between the pM9-ARF3 and pMm-ARF3. Similarly, Seleznyova *et al.* [41, 42] reported that dwarfing rootstocks reduced the shoots going through node neoformation, possibly because dwarfing apple rootstocks have a higher number of shoots that terminate growth early than more vigorous rootstocks. This also explains the shorter internode lengths of the M9 seedlings.

In *A. thaliana*, auxin produces an ‘auxin maxima’ at the apex of the primordium during the growth of lateral roots, moving from the base of the primary root to the primordium interior [43]. Subsequently, for meristem generation, auxin induces cell divisions in the pericycle founder cells [44, 45]. The ARFs are essential for transferring auxin signals to pattern formation in these cases. A previous study revealed significantly fewer emerged lateral roots in the mutant *arf3–2* seedlings than in Col-0 seedlings, implying that ARF3 plays a significant part in lateral root development [46]. Nonetheless, the portrayal of ARF3 in different development stages of lateral roots remains unclear. In apple plants, microscopy revealed that ARF3 was significantly expressed at the late stage of lateral root growth but poorly expressed at the early stage (Fig. 7A). Du and Scheres [47] divided lateral root outgrowth and emergence into seven main stages, but further investigation is needed to determine at which stage ARF3 specifically expresses during lateral root development.

LBD16 is an important transcription factor for transcriptional activation during lateral root initiation and one of the well-studied downstream targets of ARF7 and ARF19 in *A. thaliana* [26, 34]. It is present in the primordium at earlier stages [48]. In apple plants, *LBD16–2* is primarily expressed in the early stage of LRP development (Fig. 7B), consistent with in *A. thaliana*. However, unlike ARF7 and ARF19, ARF3 inhibited *LBD16–2* expression at the late-stage development of lateral root in apple plants. Therefore, we deduced that there is a special mechanism in apple plants in which ARF7 and ARF19 promote the expression of *LBD16* during the early stage of lateral root primordial development to complete the initiation of lateral root primordial. Notably, ARF3 inhibits *LBD16* expression at the late stage of lateral root primordial formation, terminating the lateral root primordia development and transforming them into the elongation stage of lateral root development.

WOX4 is primarily expressed in cambium and procambium in *A. thaliana.* RNA interference-induced down-regulation of WOX4 in Arabidopsis generated small plants whose vascular bundles accumulated undifferentiated ground tissue and exhibited severe reductions in differentiated xylem and phloem [25]. During secondary growth, BES1 and WOX4 antagonistically regulate bidirectional activity of cambial cell, during which WOX4 promotes cell proliferation while BES1 represses WOX4 transcription to promote xylem and phloem differentiation [49]. On the contrary, In PttWOX4a/b RNAi trees, primary growth was not affected whereas the width of the vascular cambium was severely reduced and secondary growth was greatly diminished [50, 51]. *MdWOX4-1* may have dual functions in apple plants. Before the lateral root emergence, *MdWOX4-1* promotes the expression of *MdLBD16-2*, promoting lateral root development. In the stage of lateral root elongation, *MdWOX4-1* promotes the expression of *MdARF3*, which inhibits the expression of *MdLBD16-2* and accumulates moderate LBD. This ends the lateral root growth and induces their elongation phase. This is the underlying cause for the observed increase in lateral root length in vigorous rootstocks (Fig. 7E).

However, in M9 seedlings, *MdWOX4–1* does not promote the expression of *ARF3* at the late development stage of lateral root owing to the loss of the WUS element on the *ARF3* promoter. Thus, the process of lateral root development could not be completed in time, which inhibited the lateral root elongation and induced the development of more short lateral roots (Fig. 7E). Based on this, *ARF3* may serve as a molecular switch between lateral root initiation and elongation. Furthermore, the short lateral roots may be the important cause of the dwarfing phenotype of M9 seedlings.

## Materials and methods

### Plant materials and cultivation conditions

A total of 24 dwarfing rootstocks and 23 vigorous rootstocks were uniformly potted in sterilized fine river sand and cultivated in a greenhouse with daytime and nighttime temperatures of 25 ± 2°C and 17 ± 2°C, respectively, with 16 hours of light [52]. The names of rootstocks can be found in Table S2 (see online supplementary material).

Apple calli ‘Orin’ were cultured in 4.43 g·L^−1^ Murashige and Skoog (MS, PhytoTechnology, M519) medium supplemented with 1.5 mg·L^−1^ of 2,4-dichloro phenoxy acetic acid (2,4-d), 0.4 mg·L^−1^ 6-benzyl amino purine (6-BA), 30 g·L^−1^ sugar, and 7.5 g·L^−1^ agar with pH adjusted to 5.8. The calli were subcultured every 20 days and incubated at 24 ± 2°C in the dark [53].

Additionally, *Nicotiana tabacum c*v. Xanthi was cultured in MS medium and incubated at 24 ± 2°C with 16 hours of light and 8 hours of darkness. When the tobacco seedlings had produced four leaves, the stem tips were cut off and transferred to new MS media for rooting experiments. The seedlings were then transplanted into a rooting medium (peat and vermiculite in a ratio of 1:1) after 20 days. After 30 days of growth in the peat and vermiculite mixture, the transgenic and wild type (WT) *N. tabacum* plants were used to determine the aboveground phenotypes. Subsequently, the T1 seeds were collected for further observation of the root phenotypes after two months of culture. All the seeds were first soaked in 70% ethanol for 30 s, then washed in three changes of ddH_2_O, followed by soaking in 2.5% sodium hypochlorite for 10 min, then washed in three changes of ddH_2_O again, and finally sown on MS medium. After three days of vernalization in the dark at 4°C, the seeds were transferred to a light incubator at 24°C with 16 hours of light and 8 hours of darkness [54]. After five days of growth, seedlings of the same size were transferred to new MS media. The root system growth was observed after 10 d.

*M. baccata* seeds were refrigerated at 4°C for a month. The germinated seeds were transferred into the soil for about 10 d. Subsequently, rooting was induced by the *A. rhizogenes* method, and transgenic apple roots were obtained after 30 d [55]. The obtained transgenic seedlings were cultured in Hoagland nutrient solution.

### Transformation and molecular confirmation of transgenic plants

*MdWOX4–1*, *MdWOX13–1,* 35S::MdARF3, and the upstream promoter with *MdARF3* coding sequences were cloned and inserted into pRI101-eGFP to construct a gene overexpression vector. *MdARF3* was also constructed into the RNAi vector. The obtained plasmids were inserted into *Agrobacterium tumefaciens* strain GV3101 to induce transformation in apple calli [56]. Subsequently, the tobacco leaf discs were modified as described by Zheng *et al.* [57].

Virus-induced gene silencing was performed as described by Chen *et al.* [58]. To create the pTRV2-MdWOX4–1 and TRV2-MdWOX13–1 vectors, 288 bp-long definite fragments of the MdWOX4–1 and MdWOX13–1 sequences, respectively, were amplified from the apple genome. Next, pTRV1, pTRV2, pTRV2-MdWOX4–1, and pTRV2-MdWOX13–1were transformed into *A. tumefaciens* strain GV3101 before introducing them in the plants, as described by Fan *et al.* [54].

Transgenic plants were generated using the Golden Delicious apple cultivar. The transformation of apple leaf explants was carried out as described by Dai *et al.* [59]. Next, the apple seedlings were micropropagated on the MS medium for 30 days before they were transferred to 1/2 MS with 0.5 mg/L indolebutyric acid to the root. After 50 days, the plants were transferred to the soil to assess the plant phenotypic characteristics.

The transgenic plants were confirmed by quantitative PCR (qPCR) using primers listed in Table S1 (see online supplementary material). The qPCR analyses were also used to measure *MdWOX4–1*, *MdWOX13–1*, and *MdARF3* gene expression levels in all transgenic lines and control plants.

### Microscopy and histochemical GUS assays

A few alterations were made to Nowicka [60] protocols of GUS staining. Briefly, the ARF3 promoters from M9 (pM9) and *M. micromalus* (pMm) were PCR-amped from genomic DNA. The element sequence of TATABOX was mutated TATTAAT into AAAAAAA. The element sequence of CACTFPPCA1 was mutated CACT to AAAA. The element sequence TTAATGG of WUSATAg was mutated into AAAAAAA. Next, mutated fragments of the A1-A6 constructs were generated using overlap PCR and transferred into the pCambia1391 vector to generate M9, Mm, and A1-A6 constructs. The eight promoter sequences were separately fused with the β-glucuronidase (GUS) coding sequence. Subsequently, the green fluorescent protein (GFP) mediated by the CaMV 35S promoter was transiently co-expressed in each tobacco (*N. tabacum* cv. Xanthi) leaf tissue. Real-time quantitative reverse transcription (qRT-PCR) was used to determine the expression of GUS and GFP in the tobacco leaves. After 24 and 48-hour dark and light treatments, respectively, leaf samples from eight injection combinations were taken with a punch. The samples were stained for 48 h at 37°C in GUS buffer. After staining, chlorophyll was removed from the samples by maintaining the samples in 75% ethanol for 24 hours. The same method was used to stain the apple roots transformed with pMmARF3: GUS or pMdLBD16–2: GUS to observe the ARF3 and LBD16–2 location during the root development process. All samples were visualized and imaged using an OLYMPUS sz61 body microscope.

The root fluorescence was detected with confocal microscopy (TE2000-E). The cell length measurement was performed using software image J.

### WGA488 fluorescent labeling

The tobacco root cells were observed using the cell membrane labeling tool AlexaFluor 488 wheat germ agglutinin (WGA488). Briefly, the roots were boiled in 10% (w/v) KOH solution for 10 min. Next, the roots were washed in three changes of Phosphate Buffered Saline (PBS) buffer and incubated in 0.2 μg/mL WGA488 in PBS overnight at 4°C. Finally, the root florescence was detected by confocal microscopy (TE2000-E) and the cell length measurement was performed using ImageJ software.

### Paraffin-embedment and histological evaluation

The 35S::MdARF3 transgenic apple plants root system was separated and treated with formaldehyde/ethanol/acetic acid solution at the root tip, about 1 cm. Next, tissue sections were prepared at Wuhan Servicebio Technology Co., Ltd. Finally, the light microscope was used for the histological evaluation of the sections.

### Determination of the cell wall substance content and measurement of phenotypic indicators

The content of three main cell wall substances, including pectin, cellulose, and hemicellulose was determined by visible spectrophotometry using the kit according to the manufacturer’s instructions. At the same time, the plant height, length of internode and root length were measured using a tape measure.

### Determination of photosynthetic indicators

Indicators of photosynthetic activity were assessed as described by Zhou *et al.* [61].

### Dual-luciferase assays

The ARF3 promoters from M9 (pM9-ARF3), *M. micromalus* (pMm-ARF3), and pMm-WUS-double were cloned upstream of the firefly LUC gene in the vector pGreenII 0800-LUC (reporter vector). The full-length CDSs of *MdARF3*, *MdWOX4-1,* or *MdWOX13-1* were cloned into the pGreenII 62-SK (effector vectors). The activity and infection assay was carried out as previously described by Wei *et al.* [62]. Finally, the fluorescence signals were detected using Night SHADE LB 985 (Berthold Technologies, Wilbad, Germany).

### Yeast Y1-H assays

The Y1-H tests were carried out according to Xie *et al.*’s instructions [63]. Briefly, ARF3 and WOX members’ CDS sequences were introduced to the pJG4-5 vector, containing the ARF3 and LBD16-2 promoters. Finally, the yeast EGY48 strain that expressed promoters-placzi and genes-pJG4-5 was cultured on the selective medium SD/His-Trp with X-Gal.

### Electrophoretic mobility shift assays (EMSA)

A full-length cDNA fragment of MdARF3 was inserted into pet32a (+) vector (His tag), while MdWOX4–1 was fused into pGex-4 T-1 vector (GST tag). The plasmids were transformed into *Escherichia coli* strains BL21 (DE3). MdARF3-His or MdWOX4–1-GST protein was induced using 0.5 mM isopropyl β-D-1-thiogalactopyranoside (IPTG). Induction was at 16°C in a shaker for 16 h. Finally, the proteins were detected by a western blot with His antibodies (CoWin Biosciences, Cambridge, MA, USA) and glutathione S- transferase (GST) as previously described [64]. The EMSA assay was performed referring to Feng *et al.*’s protocol [65], using the Light Shift Chemiluminescent EMSA Kit (Thermo Fisher Scientific, Waltham, MA, USA). All the probe sequences used in this assay are listed in Table S1 (see online supplementary material).

### Chip analysis

The ChIP analysis was carried out as previously described by Cai *et al.* [66]. ChIP DNA from 35S: MdWOX4-GFP transgenic calli was reverse cross-linked and purified with a PCR purification kit (EpiQuikTM Plant ChIP Kit, Base Catalog # P-2014).

### RNA extraction

The total RNA was extracted from transgenic tobacco and apple plants, M9 root system and *M. micromalus* using the CTAB technique [67]. Subsequently, a HiScript 1st Strand cDNA Synthesis Kit (Vazyme, Nanjing, China) was used to generate the first strand complementary DNA (cDNA).

### DNA extraction

The apple rootstock leaves were frozen in liquid nitrogen, and then ground to powder. Finally, the DNA was extracted from the ground samples according to the method of Edwards *et al.* [68].

### InDel primer design

An InDel gene marker was designed at both ends of the missing fragment to assess the 62 bp deletion sequence correlation between dwarfing and vigorous rootstocks. It was designed to only amplify the corresponding PCR product for the characteristic fragment with specific insertions or deletions. A double-band PCR product was obtained with the deletion/insertion genotype when the deletion variation site existed in the DNA template. The marker primer pairs used to identify the 24 dwarfing rootstocks and 23 vigorous rootstocks are listed in Table S3 (see online supplementary material).

### Prediction of the transcription factor binding site

Online predictions were made using the Plant Cis-acting Regulatory DNA Elements (PLACE, www.dna.affrc.go.jp/PLACE) database.

### Real-time quantitative reverse transcription PCR (qRT-PCR) analysis

The qRT-PCR analysis was performed using SYBR® Premix Ex Taq (TaKaRa, Shiga, Japan) on the QuantStudio 6 Flex Real-time PCR machine (Applied Biosystems, Waltham, MA, USA) [69]. For each treatment, three biological replicates were employed. Table S1 (see online supplementary material) lists the primers used for the gene expression analysis.

### Statistical analysis

Each experiment was performed in triplicates. Comparisons between two groups were performed by Student’s *t*-test and the ordinary one-way analysis of variance (ANOVA) for comparisons between three groups or more. The differences between the means were separated using ^*^ at ^*^P < 0.05, ^**^P < 0.01, ^***^P < 0.001, and ^****^P < 0.0001 significance levels.

## Supplementary Material

Web_Material_uhae051

## Data Availability

The manuscript and supplementary materials provide all the necessary information.
