# Supplementary material for: MdARF3 switches the lateral root elongation to regulate dwarfing in apple plants
Source: Hortic Res. 2024 Feb 23;11(4):uhae051. doi: 10.1093/hr/uhae051 (PMC11069427; doi:10.1093/hr/uhae051)
Supplement: Web_Material_uhae051 [file web_material_uhae051.docx]

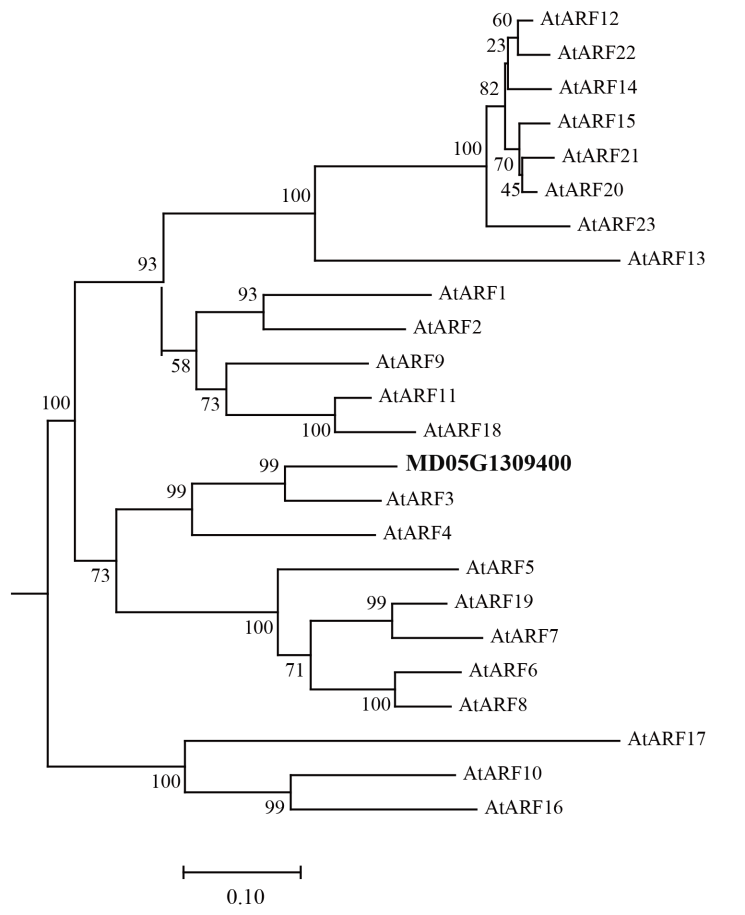


Figure S1. Maximum likelihood tree of the ARF family members contain *MD05G1309400* (marked with bold font) and *Arabidopsis thaliana.* Numbers indicate bootstrap support for the branches.


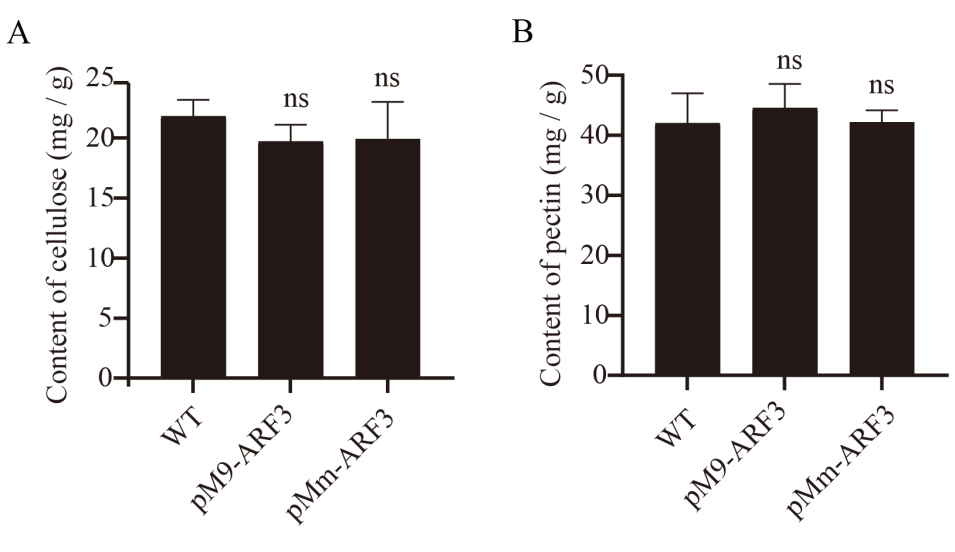


Figure S2. The content of cell wall components among the WT, pM9-ARF3 and pMm-ARF3 transgenic tobacco root. A. The content of cellulose in three type tobacco root. B. The content of pectin in three type tobacco root. ns, no significantly difference.


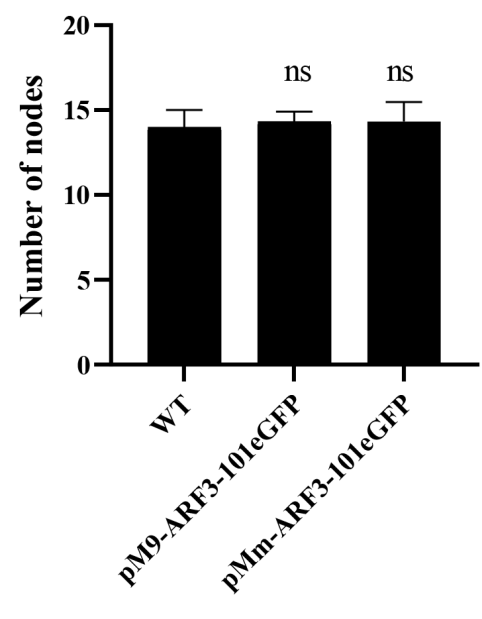


Figure S3. Number of nodes in the WT, pM9-ARF3 and pMm-ARF3 transgenic tobaco. ns, no significantly difference.


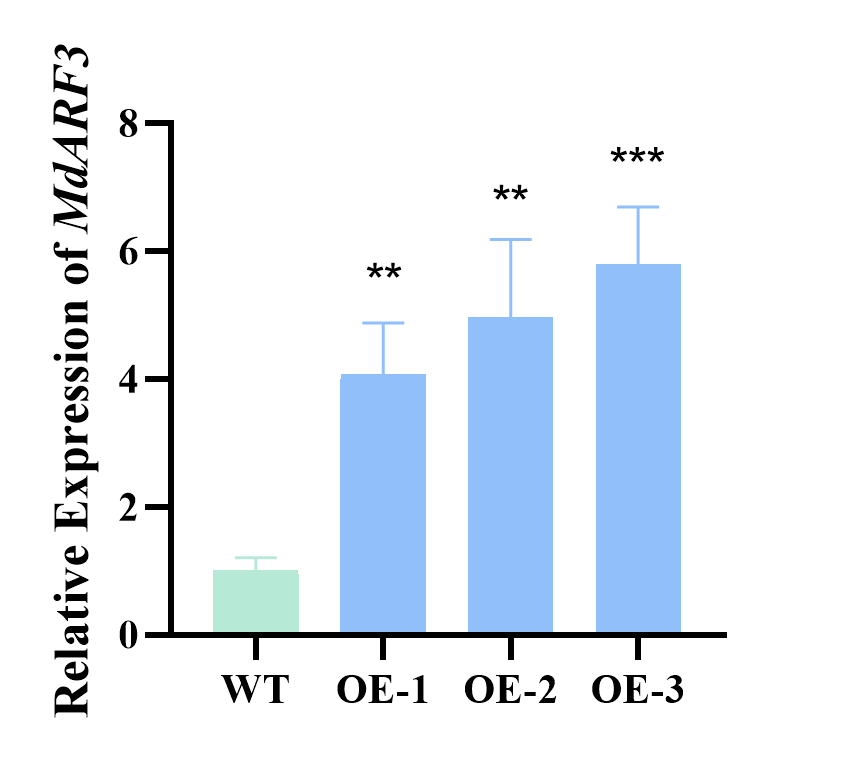


Figure S4. Relative expression of MdARF3 in WT and 35S:MdARF3 transgenic apple. ***p*<0.01.****p*<0.001.


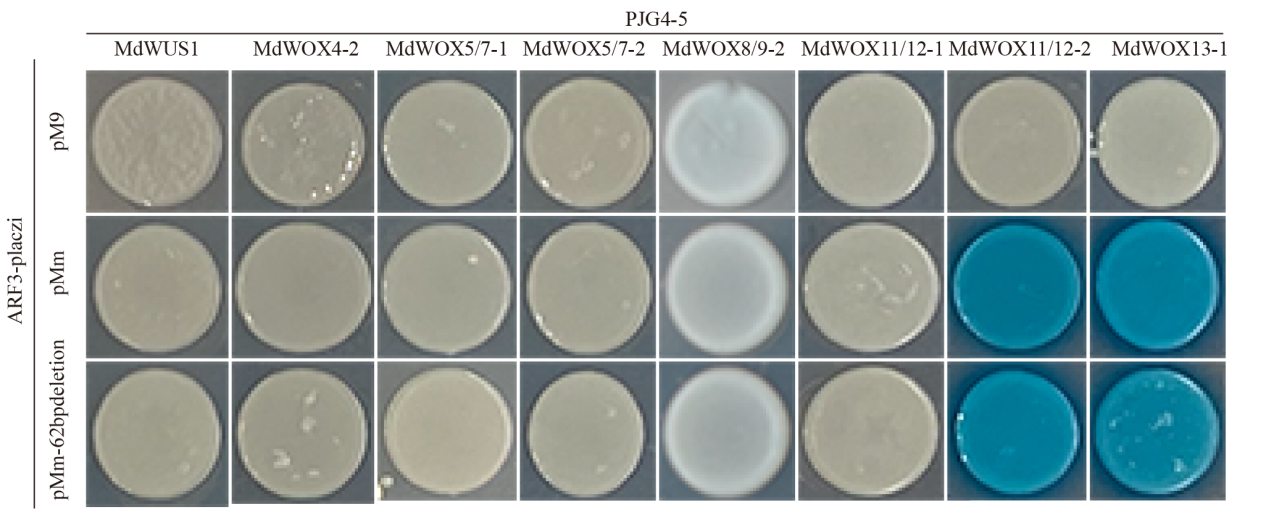


Figure S5 Y1H assay of the interaction between MdWOXs and the promoter of pM9-ARF3, pMm-ARF3 and pMm-62bp deletion, respectively. The yeast EGY48 strain expressing pM9-ARF3-placZi, pMm-ARF3-placZi, pMm-ARF3-62bp deletion-placZi, and MdWOXs-pJG4–5 grew on the selective medium SD/His-Trp with X-Gal. Blue means interaction. White means no interaction.


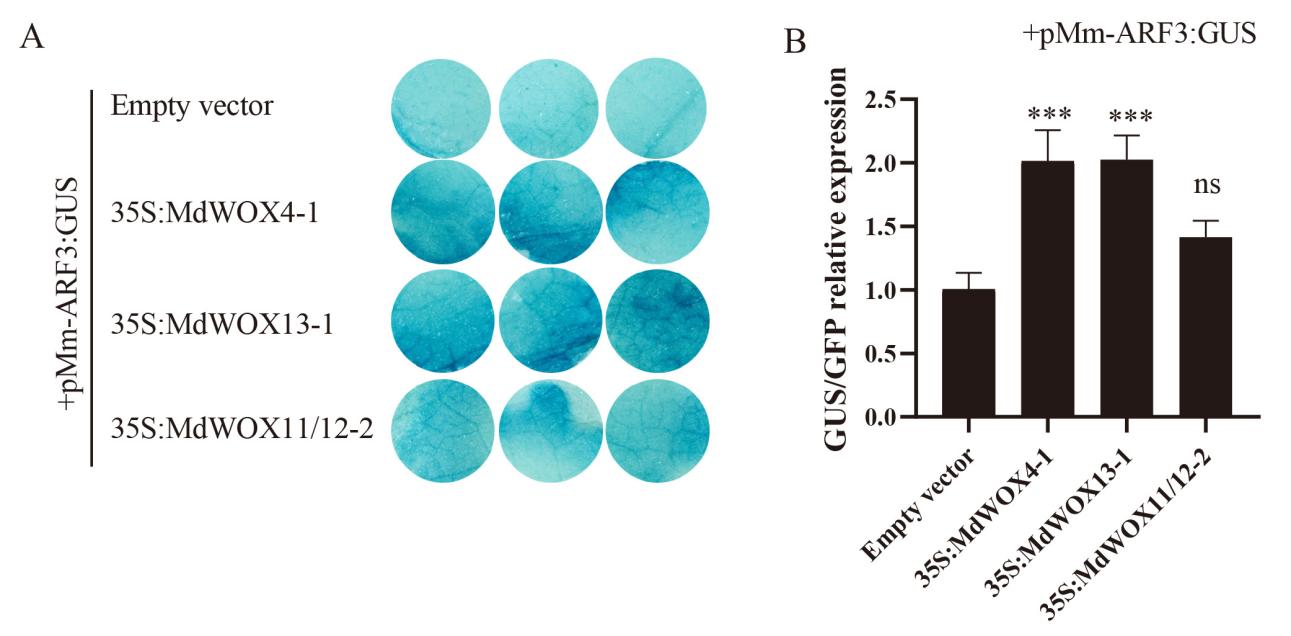


Figure S6 WOXs enhances GUS activity of pMmARF3 promoter. A. GUS staining of transiently co-expressed combinations of pMm-ARF3:GUS and 35S:MdWOXs (35S:MdWOX4-1, 35S:MdWOX13-1, 35S:MdWOX11/12-1 respectively) or Empty vector, and pRI101-eGFP (as an internal parameter factor) in tobacco leaf. B. GUS/GFP relative expression in transiently co-expressed combinations of tobacco leaf. ns, no significantly difference; *** *P* <0.001.


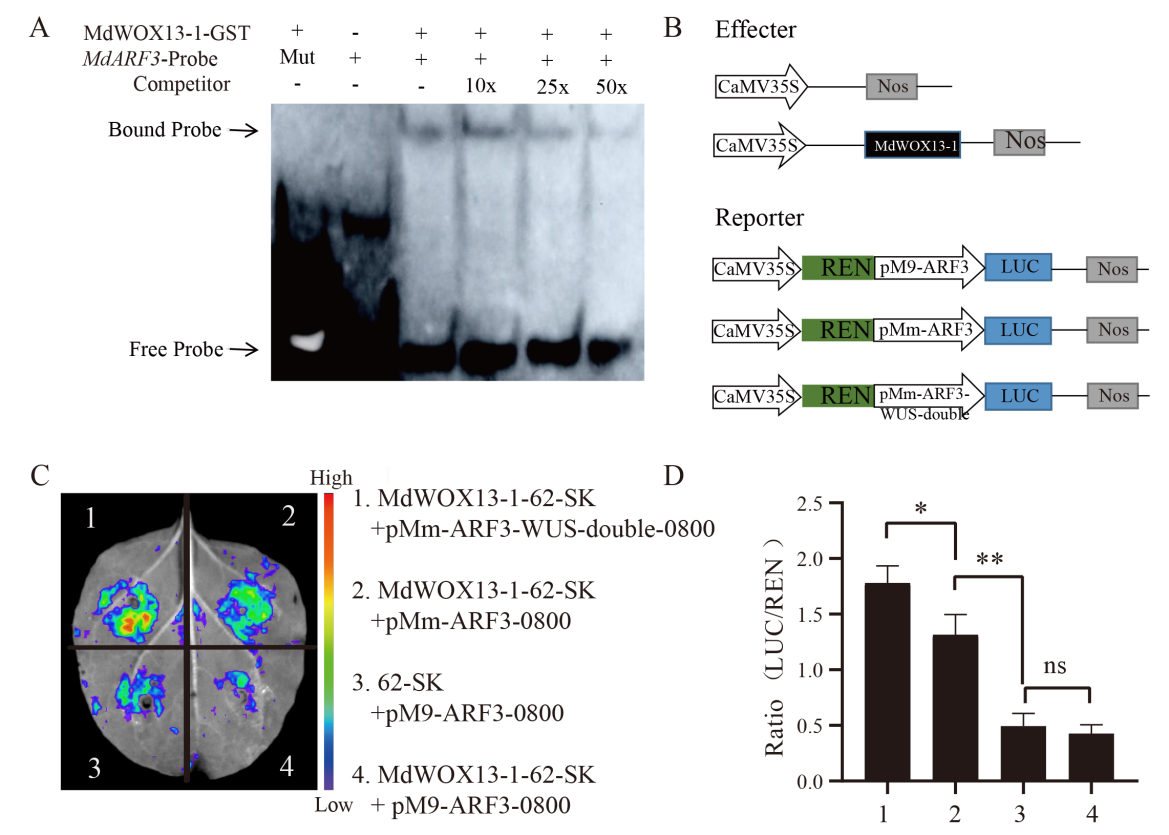


Figure S7. MdWOX13-1 promotes ARF3 expression by binding WUS elements in vitro. A. EMSA showing that MdWOX13-1 binds to the ARF3 promoter. “-” indicates the absence of corresponding of proteins or probe. “+” indicates the presence of corresponding proteins or probes. “10x” , “25x” and “50x” indicate unlabeled probes were used as competitors. B. Schematic diagrams of effector and reporter constructs used for the dual-luciferasee (LUC) sssay. C. Imaging analysis of the dual-luciferasee. Red color represents stronger signal and violet color represents weaker signal. D. Effect of MdWOX13-1 on the regulation of the ARF3 promoter in tobacco leaves and LUC/REN ratio analysis. ns, no significantly difference; **p*<0.05; ***p*<0.01.


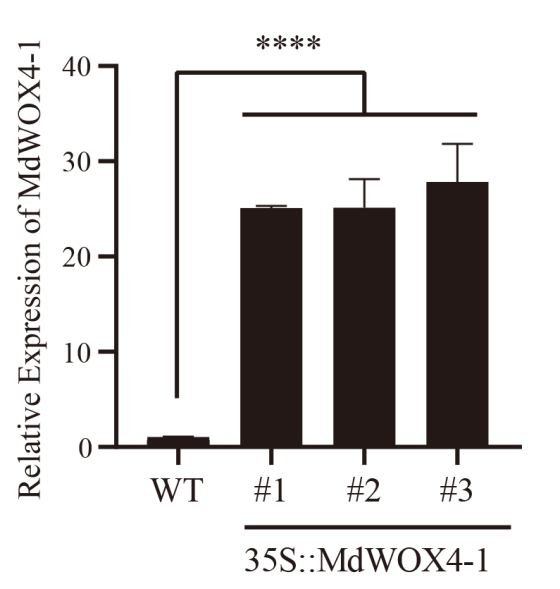


Figure S8. Overexpression of MdWOX4-1 transgenic callus identification RNA level identification. There are three lines, #1, #2, #3. ****p*<0.001.


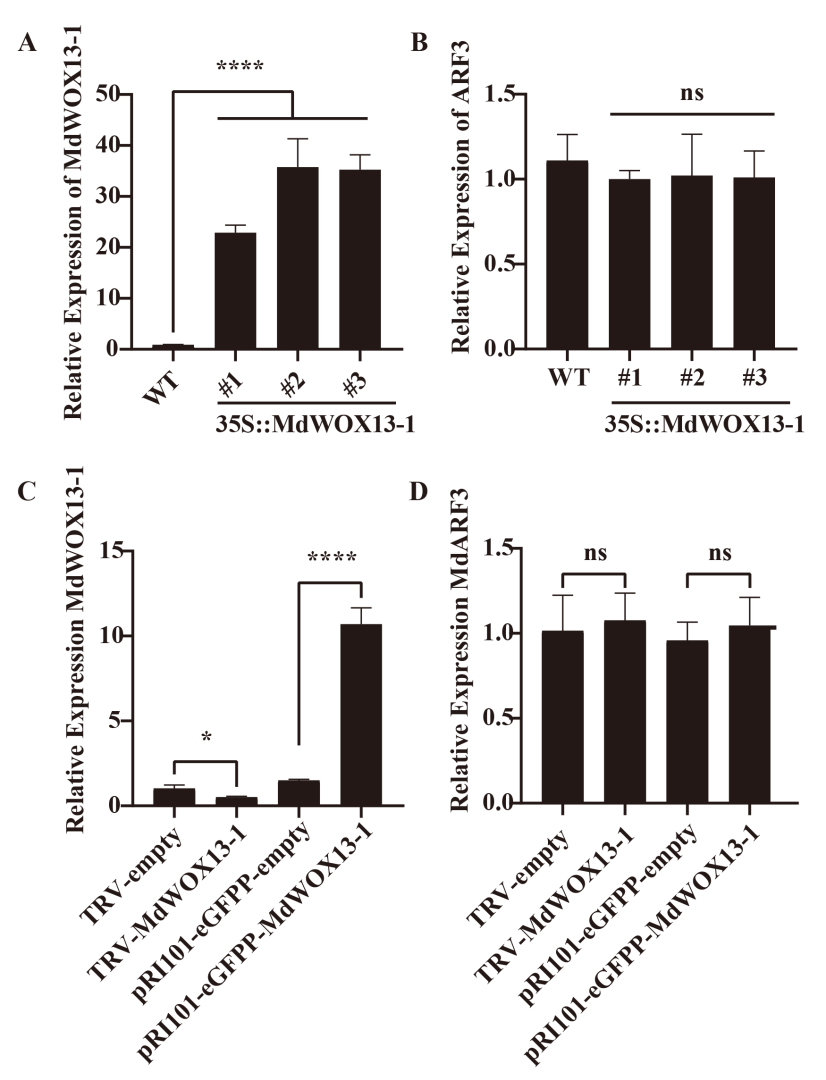


Figure S9. MdARF3 was not regulated by MdMOX13-1 in apple. A. Identification of 35S::MdWOX13-1 transgenic callus in RNA level. B. Relative expression of ARF3 in MdWOX13-1 transgenic callus. C. Identification of pRI101-eGFP-MdWOX13-1 and TRV-MdWOX13-1 transgenic apple roots in RNA level. D. Relative expression of ARF3 in MdWOX13-1 transgenic apple roots. Error bars indicate standard deviations. ns, no significantly difference; **p*<0.05; *****P*<0.0001.


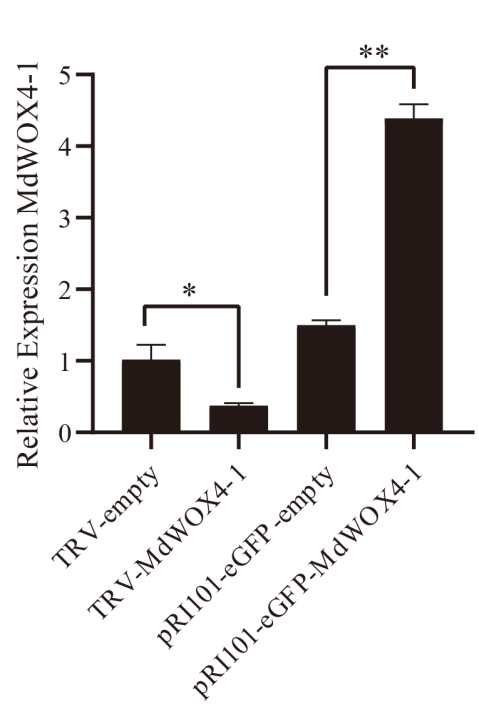


Figure S10. The identification of MdWOX4-1 in stantaneous transformation of apple roots. ***p*<0.01.


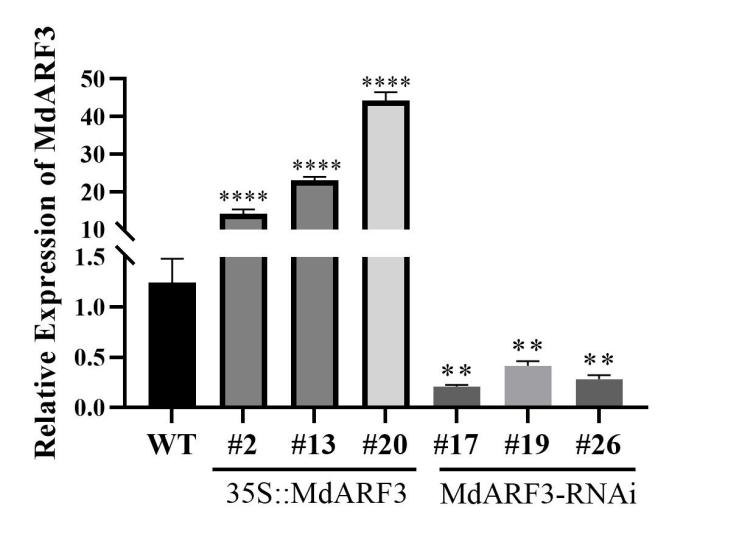


Figure S11. The relative expression level of MdARF3 in three kinds of apple callus (WT, 35S::MdARF3, MdARF3-RNAi). Values represent the mean ± SE of three biological replicates.


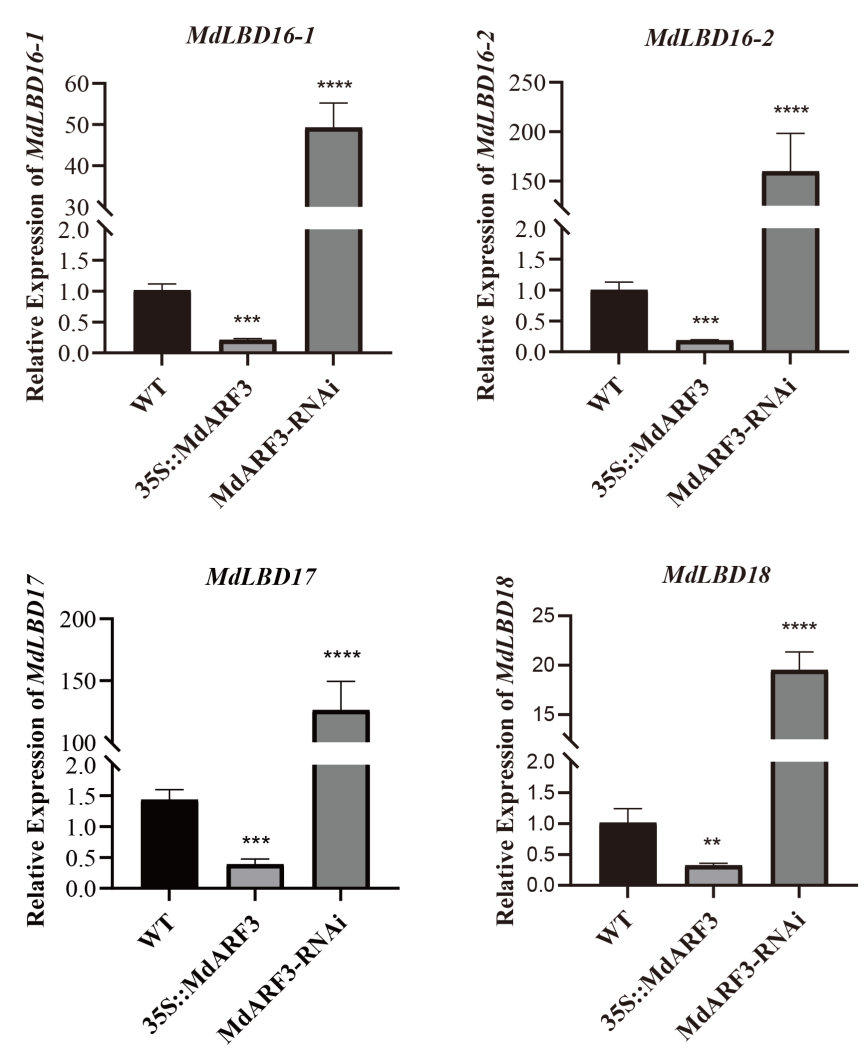


Figure S12. MdARF3 down-regulates the expression of MdLBDs. The relative expression level of MdLBD16-1, MdLBD16-2, MdLBD17, MdLBD18 in three kinds of apple callus. (WT, 35S::MdARF3, MdARF3-RNAi). ***p*<0.01; ****P*<0.001; *****P*<0.0001.


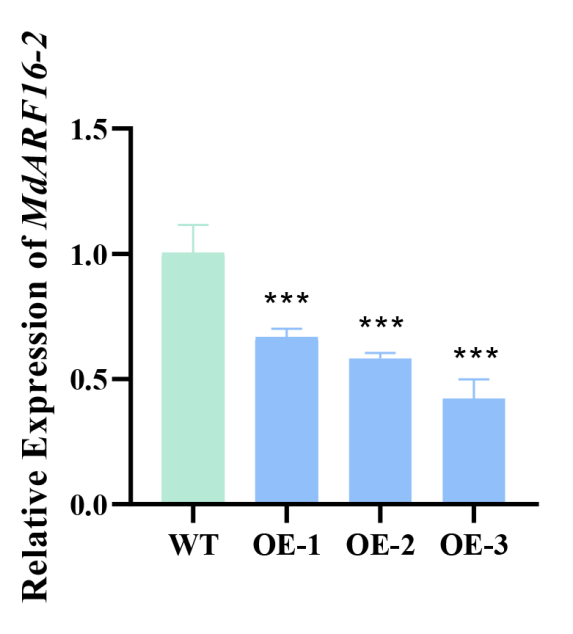


Figure S13. The relative expression level of *MdLBD16-2* in 35S::MdARF3 transgenic apple plants. ****P*<0.001.


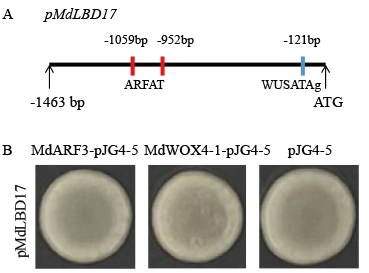


Figure S14. Yeast one hybrid test showed that neither MdARF3 nor MdWOX4-1 could bind to the promoter of MdLBD17. A. The examination of the MdLBD17 promoter's cis-element. B. Yeast cell MdARF3 and MdWOX4-1 transcriptional activation. The yeast EGY48 strain that expressed pMdLBD16-2-placzi and MdARF3-pJG4-5 or MdWOX4-1-pJG4-5 grew on the selective medium SD/His-Trp with X-Gal.


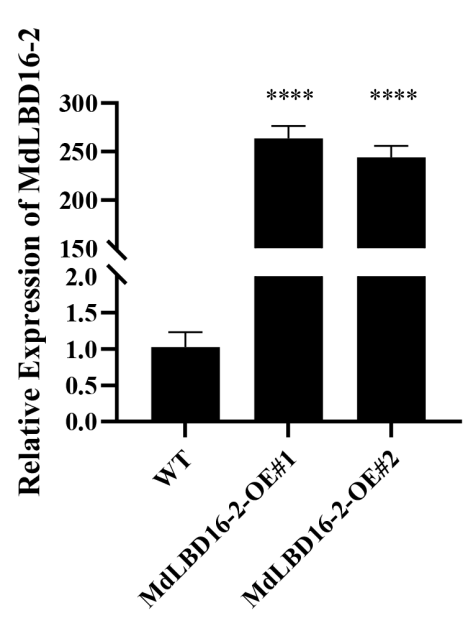


Figure S15. Overexpression of MdLBD16-2 transgenic tobacco identification RNA level identification. There are two lines, #1, #2. ****p*<0.001.


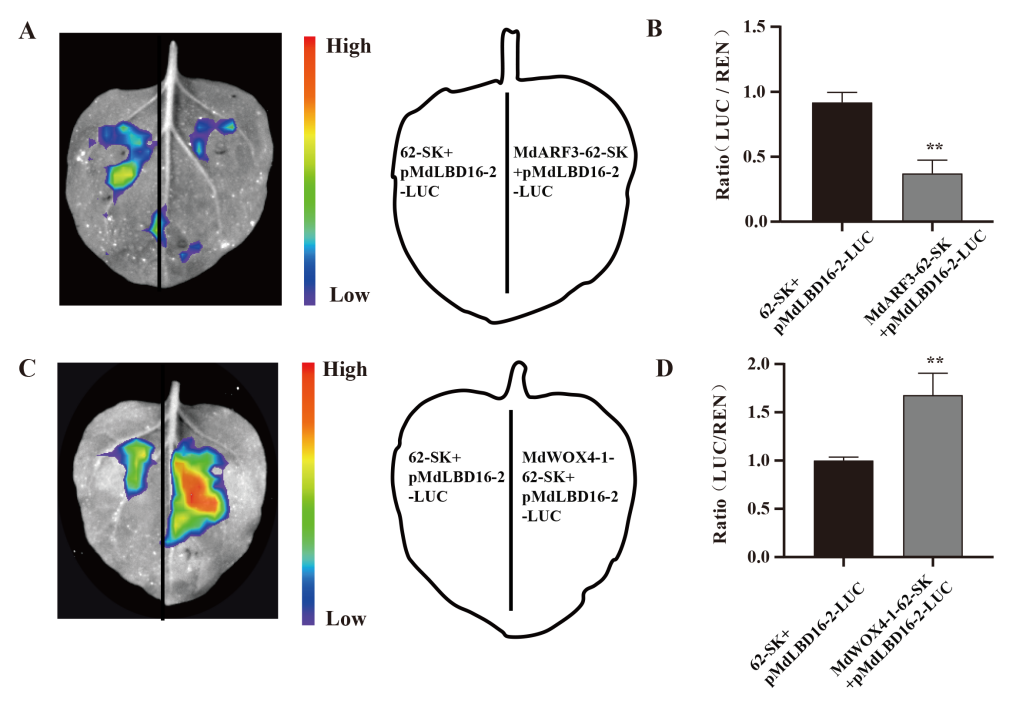


Figure S16. LUC/REN Analysis the effect of MdARF3 or MdWOX4-1 on the control of the *MdLBD16-2* promoter in tobacco leaves . A. C. Imaging analysis of the dual-luciferasee. Red color represents stronger signal and violet color represents weaker signal. B. D. Effect of MdARF3 and MdWOX4-1 on the regulation of the MdLBD16-2 promoter in tobacco leaves and LUC/REN ratio analysis.A stronger signal is represented by red, whereas a weaker signal is represented by violet..***p*<0.01.

Table S1 Primers information used in this study

| Primer name | Primer sequence |
| --- | --- |
| ARF3-qPCR-F | GCCTGGCTTTAGGAATGG |
| ARF3-qPCR-R | CACCGAATGAGGATGATGTTTGC |
|  |  |
| ARF3-PJG4-5-EcoR1-F | GCCAGATTATGCCTCTCCCGAATTCATGGCGGGTCTAATTGATCTGAA |
| ARF3-PJG4-5-EcoR1-R | GCTTCTCGAGTCGGCCGAATTCTCATGCAGCAAAGAGGTTGCTTA |
|  |  |
| ARF3-PLAZI-Sal1-F | GGTACCCGGGGATCTGTCGACTGGATTTTGTGGGATTTGAAAGCAAG |
| ARF3-PLAZI-Sal1-R | AGCACATGCCTCGAGGTCGACGTGAAATTAAACAGAAACTGTAAGG |
|  |  |
| ARF3-62SK-Bamh1-F | GCTCTAGAACTAGTGGATCCATGGCGGGTCTAATTGATCTGAA |
| ARF3-62SK-Bamh1-R | TCCTGCAGCCCGGGGGATCCTGCAGCAAAGAGGTTGCTTA |
|  |  |
| ARF3-0800-Bamh1-F | GATATCGAATTCCTGCAGCCCGGGGGATCCTGGATTTTGTGGGATTT  GAAAGCAAG |
| ARF3-0800-Bamh1-R | GCGGCCGCTCTAGAACTAGTGGATCCGTGAAATTAAA  CAGAAACTGTAAGG |
|  |  |
| ARF3-Pet32a-BamH1-F | TTGTCGACGGAGCTCGAATTCGGATCCATGGCGGGTCTAATT  GATCTGAA |
| ARF3-Pet32a-BamH1-R | GACAAGGCCATGGCTGATATCGGATCCTGCAGCAAAGAGGTTGCTTA |
|  |  |
| ARF3-Bio-F | TTAATAGTGTTAATGGTCTGTGTAGTCTAA |
| ARF3-Bio-R | TTAGACTACACAGACCATTAACACTATTAA |
|  |  |
| ARF3-Bio-mut-F | TTAATAGTGAAAAAAATCTGTGTAGTCTAA |
| ARF3-Bio-mut-R | TTAGACTACACAGACCCTTAACACTATTAA |
|  |  |
| pARF3-62bp-deletion-A1-F1-1391 | TGTTGGGCCCGGCGCGCCAAGCTTTGGATTTTGTGGGATTTGAA |
| pARF3-62bp-deleton-A1-R1-1391 | CACTATTAATTCTTATTAAAACATAAT |
|  |  |
| pARF3-62bp-deletion-A1-F2-1391 | TCTGTGTAGTCTAATGACACTTATTG |
| pARF3-62bp-deletion-A1-R2-1391 | GATCCGTCGACCTGCAGCCAAGCTTGTGAAATTA  AACAGAAACTGTAAGG |
|  |  |
| pARF3-62bp-marker-F | GACGATATCAATTAGATTGATGATGTC |
| pARF3-62bp-marker-R | CATGTCGTGGTTGTGTCTCT |
|  |  |
| pARF3-TATABOX-mut-F | AAAAAAAGTGTTAATGG |
| pARF3-TATABOX-mut-R | TTTTTTTTCTTATTAAAAC |
|  |  |
| pARF3-CACT-mut-F | CTGTGTAGTCTAAGGACAAA |
| pARF3-CACT-mut-R | TTTGTCCTTAGACTACACAG |
|  |  |
| pARF3-WUS-mut-F | TAATAGTGAAAAAAATCTGT |
| pARF3-WUS-mut-R | ACAGATTTTTTTCACTATTA |
|  |  |
| pARF3-WUS-double-F | TAATAGTGTTAATGGTTAATGGTCTGT |
| pARF3-WUS-double-R | ACAGACCATTAACCATTAACACTATTA |
|  |  |
| MdWOX4-1-PJG4-5-Ecor1-F | GCCAGATTATGCCTCTCCCGAATTCATGGGAATGAGCAGCA |
| MdWOX4-1-PJG4-5-Ecor1-R | GCTTCTCGAGTCGGCCGAATTCTCATCTTCTTCCTTCTGGAT |
|  |  |
| MdWOX4-1-62SK-Bamh1-F | GCTCTAGAACTAGTGGATCCATGGGAATGAGCAGCA |
| MdWOX4-1-62SK-Bamh1-R | TCCTGCAGCCCGGGGGATCCTCTTCTTCCTTCTGGAT |
|  |  |
| MdWOX4-1-4T-1-BamH1-F | ATCGGATCTGGTTCCGCGTGATGGGAATGAGCAGCA |
| MdWOX4-1-4T-1-BamH1-R | GTCGACCCGGGAATTCCGGGTCATCTTCTTCCTTCTGGAT |
|  |  |
| MdWOX4-1-qPCR-F | CCAGAATCACAAGGCACG |
| MdWOX4-1-qPCR-R | TTCCACCACTACTTCTCCCAG |
|  |  |
| MdWOX4-1-1300MYC-kpn1-F | GACATTTAAATACTAGTGGATCCGGTACCATGGGAATGAGCAGCA |
| MdWOX4-1-1300MYC-kpn1-R | GAAATGAGCTTTTGCTCCATGGTACCTCTTCTTCCTTCTGGAT |
|  |  |
| MdWOX4-TRV2-Xba1-F | TGAGTAAGGTTACCGAATTCTCTAGACCAGAATCACAAGGCACG |
| MdWOX4-TRV2-Xba1-R | GGATCCCCATGGAGGCCTTCTAGACTTCTTCCTTCTGGATGTAATGG |
|  |  |
| MdWOX13-1-F-PJG4-5 | GCCAGATTATGCCTCTCCCGAATTCATGATGGGGGAGTGG |
| MdWOX13-1-R-PJG4-5 | GCTTCTCGAGTCGGCCGAATTCTTACCAATCAAAATTTCCAAATC |
|  |  |
| MdWOX13-1-4T-1-BamH1-F | ATCGGATCTGGTTCCGCGTGATGATGGGGGAGTGG |
| MdWOX13-1-4T-1-BamH1-R | GTCGACCCGGGAATTCCGGGTTACCAATCAAAATTTCCAAATC |
|  |  |
| MdWOX13-1-62SK-F-WF | GCTCTAGAACTAGTGGATCCATGATGGGGGAGTGG |
| MdWOX13-1-62SK-R-WF | TCCTGCAGCCCGGGGGATCCCCAATCAAAATTTCCAAATC |
|  |  |
| MdWOX13-1-1300MYC-kpn1-F | GACATTTAAATACTAGTGGATCCGGTACCATGATGGGGGAGTGG |
| MdWOX13-1-1300MYC-kpn1-R | GAAATGAGCTTTTGCTCCATGGTACCCCAATCAAAATTTCCAAATC |
|  |  |
| MdWOX13-1-TRV2-Xba1-F | TGAGTAAGGTTACCGAATTCTCTAGAGAGGAAGGCAACGGAACT |
| MdWOX13-1-TRV2-Xba1-R | GGATCCCCATGGAGGCCTTCTAGACAAATCCTAATTCCGGGCT |
|  |  |
| MdWUS-2-F-PJG4-5 | GCCAGATTATGCCTCTCCCGAATTCATGGACCCTCAACAAAAC |
| MdWUS-2-R-PJG4-5 | GCTTCTCGAGTCGGCCGAATTCTTAACATGATCTGAAGCAATC |
|  |  |
| MdWOX4-2-PJG-F | GCCAGATTATGCCTCTCCCGAATTCATGCATACATGTCTCTCTTTC |
| MdWOX4-2-PJG-R | GCTTCTCGAGTCGGCCGAATTCTTTTCTTCCTTCTGGATGT |
|  |  |
| MdWOX5/7-1-PJG-F | GCCAGATTATGCCTCTCCCGAATTCATGGACGAGGGCATGT |
| MdWOX5/7-1-PJG-R | GCTTCTCGAGTCGGCCGAATTCTCAAAGGAAACTTAAACGCAG |
|  |  |
| MdWOX5/7-2-PJG-F | GCCAGATTATGCCTCTCCCGAATTCATGGACGATGGCATGTCAG |
| MdWOX5/7-2-PJG-R | GCTTCTCGAGTCGGCCGAATTCATGGAAACTTAAGCGCAGATC |
|  |  |
| MdWOX8/9-1-F-PJG4-5 | GCCAGATTATGCCTCTCCCGAATTCATGGCTTCATCAAACAGA |
| MdWOX8/9-1-R-PJG4-5 | GCTTCTCGAGTCGGCCGAATTCCTATATCAGATAGTAGAAACCACC |
|  |  |
| MdWOX11/12-1-PJG4-5-F | GCCAGATTATGCCTCTCCCGAATTCATGGAAGATCATCATCAAG  GCCAAG |
| MdWOX11/12-1-PJG4-5-R | GCTTCTCGAGTCGGCCGAATTCATTTGCAAAGAACAAAATGGTTAGCGG |
|  |  |
| MdWOX11/12-2-F-PJG4-5 | GCCAGATTATGCCTCTCCCGAATTCATGGAAGATCATCATCAACATC |
| MdWOX11/12-2-R-PJG4-5 | GCTTCTCGAGTCGGCCGAATTCTTAACTTGGTCTTGAAACCAG |
|  |  |
| MdLBD16-2-qPCR-F | TTTCCTAGTGTGCTCGTCGC |
| MdLBD16-2-qPCR-R | TGAATGGCAGCAAAACGAGC |
|  |  |
| LBD16-2-Bio-F-ARF | TAGTTTTCCTTTTTGTCTCATGTGATCAATGGC |
| LBD16-2-Bio-R-ARF | GCCATTGATCACATGAGACAAAAAGGAAAACTA |
|  |  |
| LBD16-2-Bio-F-MdWOX | TAATCATACTCGGTAGCCATTAATCATCCACGC |
| LBD16-2-Bio-R-MdWOX | GCGTGGATGATTAATGGCTACCGAGTATGATTA |
|  |  |
| EF-1α-F | ATTCAAGTATGCCTGGGTGC |
| EF-1α-R | CAGTCAGCCTGTGATGTTCC |
|  |  |
| ARF3-WUS-qChip-F | GACGATATCAATTAGATTGATGATGTC |
| ARF3-WUS-qChip-R | CATGTCGTGGTTGTGTCTCT |
|  |  |
| MdARF3-101-eGFP-F | CACTGTTGATACATATGCCCctcgacATGGCGGGTCTAATTGATCTGAACA |
| MdARF3-101-eGFP-R | CATGGATCCGGTACCCCCGGGgtcgacTGCAGCAAAGAGGTTGCTTAC |
|  |  |
| MdLBD16-2-101-eGFP-F | GTTGATACATATGCCCGTCGACATGCCCCACTTTCCTCC |
| MdLBD16-2-101-eGFP-R | TGCTCACCATGGATCCGGTACCTCAGTTTCTCATCATTCTGAAGG |

| ID | Annotation |
| --- | --- |
| *MD05G1309400* | Auxin response factor 3 |
| *MD05G1306800* | Ethylene-responsive transcription factor ERF091 |
| *MD05G1292400* | ABC transporter B family member 9 |
| *MD05G1300200* | Abscisic acid receptor PYL6 |
| *MD05G1282700* | 9-cis-epoxycarotenoid dioxygenase NCED1, chloroplastic |
| *MD05G1283800* | Gibberellin 2-beta-dioxygenase |

Table S2 Screening of genes related to hormone metabolism in DW1

Table S3 Statistics of type and variation of dwarfing rootstocks and vigorous rootstocks

| **Dwarfing rootstock type that contains M9 in any parent (12)** | **Genotype** |
| --- | --- |
| M9 | deletion/insertion |
| Mac9 | deletion/insertion |
| M27 | deletion/insertion |
| M26 | deletion/insertion |
| 77-34 | deletion/insertion |
| CX5 | deletion/insertion |
| Liaozhen 2 | deletion/insertion |
| B9 | deletion/insertion |
| JM7 | deletion/insertion |
| KM23 | deletion/insertion |
| B118 | deletion/insertion |
| OT3 | deletion/insertion |
|  |  |
| **Dwarfing rootstock types that do not contain M9 in either parent (12)** | **Genotype** |
| SH6 | deletion/insertion |
| SH9 | deletion/insertion |
| SH29 | deletion/insertion |
| CG24 | deletion/insertion |
| CG80 | deletion/insertion |
| Liaozhen 109 | deletion/insertion |
| M7 | deletion/insertion |
| G30 | deletion/insertion |
| GM256 | deletion/insertion |
| CG10 | insertion/insertion |
| Zhaai76 | insertion/insertion |
| Malus asiatica Nakai in Laoshan | insertion/insertion |
|  |  |
| **Vigrous rootstock type (23)** | **Genotype** |
| Malus Micromalus 'Ba leng' | insertion/insertion |
| Malus hupehensis (Pamp) Rehder | insertion/insertion |
| Malus micromalus Makino (M.baccata x M.spectabilis) | insertion/insertion |
| Malus halliana Koehne | insertion/insertion |
| Hypericum ascyron | insertion/insertion |
| Malus kansuensis (Batal.) Schneid | insertion/insertion |
| Malus sikkimensis (Wenzig) Koehne | insertion/insertion |
| Bredia rotundifolia | insertion/insertion |
| Malus baccata (L.) Borkh | insertion/insertion |
| Malus rockii Rehd | insertion/insertion |
| Malus platycarpa Rehd | insertion/insertion |
| Malus coronaria | insertion/insertion |
| Malus prunifolia (Willd.) Borkh | insertion/insertion |
| Malus 'Royalty' | insertion/insertion |
| Malus Micromalus 'Lai wu' | insertion/insertion |
| Malus hupehensis | insertion/insertion |
| Antonov | insertion/insertion |
| Malus kansuensis (Batalin C. K. Schneid.) | insertion/insertion |
| Malus rockii Rehd | insertion/insertion |
| Malus manshurica (Maxim.) Komarov | deletion/insertion |
| Malus sieboldii (Reg.) Rehd | deletion/insertion |
| Malus 'Niu Ma Ma' | deletion/insertion |
| Malus toringoides (Rehd) Hughes | deletion/insertion |

Table S4 Element prediction of 62bp deletion promoter sequence

| Factor or Site name | Loc.(Str.) | Signal Sequence | Annotation |
| --- | --- | --- | --- |
| TATABOX3 | 25 (-) | TATTAAT | TATA box found in the 5'upstream region of sweet potato sporamin A gene |
| CACTFTPPCA1 | 31 (-) | YACT | Cis-Regulatory elements for mesophyll-specific gene expression |
| WUSATAg | 35 (+) | TTAATGG | Target sequence of WUS in the intron of AGAMOUS gene in Arabidopsis |
| CACTFTPPCA1 | 59 (+) | YACT | cis-Regulatory elements for mesophyll-specific gene expression |
